# Manual-Protocol Inspired Technique for Improving Automated MR Image Segmentation during Label Fusion

**DOI:** 10.3389/fnins.2016.00325

**Published:** 2016-07-19

**Authors:** Nikhil Bhagwat, Jon Pipitone, Julie L. Winterburn, Ting Guo, Emma G. Duerden, Aristotle N. Voineskos, Martin Lepage, Steven P. Miller, Jens C. Pruessner, M. Mallar Chakravarty

**Affiliations:** ^1^Institute of Biomaterials and Biomedical Engineering, University of TorontoToronto, ON, Canada; ^2^Cerebral Imaging Centre, Douglas Mental Health University InstituteVerdun, QC, Canada; ^3^Kimel Family Translational Imaging-Genetics Research Lab, Research Imaging Centre, Campbell Family Mental Health Research Institute, Centre for Addiction and Mental HealthToronto, ON, Canada; ^4^Neurosciences and Mental Health, The Hospital for Sick Children Research InstituteToronto, ON, Canada; ^5^Department of Paediatrics, The Hospital for Sick Children and the University of TorontoToronto, ON, Canada; ^6^Department of Psychiatry, University of TorontoToronto, ON, Canada; ^7^Department of Psychiatry, McGill UniversityMontreal, QC, Canada; ^8^McGill Centre for Studies in AgingMontreal, QC, Canada; ^9^Biological and Biomedical Engineering, McGill UniversityMontreal, QC, Canada

**Keywords:** MR Imaging, segmentation, multi-atlas label fusion, hippocampus, Alzheimer's disease, first-episode-psychosis, premature birth and neonates

## Abstract

Recent advances in multi-atlas based algorithms address many of the previous limitations in model-based and probabilistic segmentation methods. However, at the label fusion stage, a majority of algorithms focus primarily on optimizing weight-maps associated with the atlas library based on a theoretical objective function that approximates the segmentation error. In contrast, we propose a novel method—Autocorrecting Walks over Localized Markov Random Fields (AWoL-MRF)—that aims at mimicking the sequential process of manual segmentation, which is the gold-standard for virtually all the segmentation methods. AWoL-MRF begins with a set of candidate labels generated by a multi-atlas segmentation pipeline as an initial label distribution and refines low confidence regions based on a localized Markov random field (L-MRF) model using a novel sequential inference process (walks). We show that AWoL-MRF produces state-of-the-art results with superior accuracy and robustness with a small atlas library compared to existing methods. We validate the proposed approach by performing hippocampal segmentations on three independent datasets: (1) Alzheimer's Disease Neuroimaging Database (ADNI); (2) First Episode Psychosis patient cohort; and (3) A cohort of preterm neonates scanned early in life and at term-equivalent age. We assess the improvement in the performance qualitatively as well as quantitatively by comparing AWoL-MRF with majority vote, STAPLE, and Joint Label Fusion methods. AWoL-MRF reaches a maximum accuracy of 0.881 (dataset 1), 0.897 (dataset 2), and 0.807 (dataset 3) based on Dice similarity coefficient metric, offering significant performance improvements with a smaller atlas library (< 10) over compared methods. We also evaluate the diagnostic utility of AWoL-MRF by analyzing the volume differences per disease category in the ADNI1: Complete Screening dataset. We have made the source code for AWoL-MRF public at: https://github.com/CobraLab/AWoL-MRF.

## Introduction

The volumetric and morphometric analysis of neuroanatomical structures is increasingly important in many clinical applications. For instance, structural characteristics of the hippocampus have been used as an important biomarker in many neuropsychiatric disorders including Alzheimers disease (AD), schizophrenia, major depression, and bipolar disorder (Harrison, 2004; Frey et al., 2007; Lerch et al., 2008; Kempton et al., 2011; Meda et al., 2013; Weiner, 2013). The gold standard for neuroanatomical segmentation is manual delineation by an expert human rater. However, with the increasing ubiquity of magnetic resonance (MR) imaging technology and neuroimaging studies targeting larger populations, the time and expertise required for manual segmentation of large MR datasets becomes a critical bottleneck in analysis pipelines (Mazziotta et al., 1995, 2001; Pausova et al., 2007). Manual rater performance is dependent on specialized knowledge of the neuroanatomy. A generic manual segmentation protocol leverages this anatomical knowledge and uses it in tandem with voxel intensities to enforce structural boundary conditions during the delineation process. This is, of course, the premise of many automated model-based segmentation approaches.

Multi-atlas based approaches have been shown to improve segmentation accuracy and precision over model-based approaches (Collins et al., 1995; Pruessner et al., 2000; Warfield et al., 2004; Heckemann et al., 2006, 2011; Aljabar et al., 2009; Chakravarty et al., 2009, 2013; Leung et al., 2010; Lötjönen et al., 2010; Sabuncu et al., 2010; Wolz et al., 2010; Wang et al., 2012; Yushkevich et al., 2012). The processing pipelines of these approaches can be divided into multiple stages. First, several atlas images are registered to a target image, i.e., an image to be segmented. Subsequently, the atlas labels are propagated to produce several candidate segmentations of the target image. Finally, a label fusion technique such as voxel-wise voting is used to merge these candidate labels into the final segmentation for the target image. For the remainder of the manuscript we refer this latter stage within a multi-atlas based segmentation pipeline as “label fusion,” which is the core interest of this work.

Traditionally, in many image processing and computer vision applications in neuroimaging, the use of Markov Random Field (MRF) has been a popular approach for modeling spatial dependencies and has been used in several model-based segmentation techniques. Existing software packages such as FreeSurfer (Fischl et al., 2002) and FMRIB Software Library (Smith et al., 2004) use MRF for gray matter, white matter, and cerebrospinal fluid classification as well as for segmentation of multiple subcortical structures. For example, FreeSurfer uses an anisotropic non-stationary MRF that encodes the inter-voxel dependencies as a function of location within the brain. Pertaining to multi-atlas label fusion techniques, STAPLE (Simultaneous Truth And Performance Level Estimation; Warfield et al., 2004), uses a probabilistic performance framework consisting of an MRF model and an Expectation-Maximization (EM) inference method to compute the probabilistic estimate of a true segmentation based on an optimal combination of a collection of segmentations. STAPLE has been explored in several studies for improving a variety of segmentation tasks (Commowick and Warfield, 2010; Akhondi-Asl and Warfield, 2012; Commowick et al., 2012; Jorge Cardoso et al., 2013).

Alternatively, a majority of modern multi-atlas approaches treat label fusion as a weight-estimation problem, where the objective is to estimate optimal weights for the candidate segmentation propagated from each atlas. In a trivial case with uniform weights, this label fusion technique boils down to a simple majority vote. In other cases (Aljabar et al., 2009), the weights can be used to exclude atlases that are dissimilar to a target image to minimize the errors from unrepresentative anatomy. In a more general case, weight values are estimated using some similarity metric between the atlas library and the target image. A comprehensive probabilistic generative framework is provided by Sabuncu et al. (2010) that models such an underlying relationship between the atlas and target data, exploited by the methods belonging to this class. More recently, several methods (Coupé et al., 2011; Rousseau et al., 2011; Wang et al., 2012) have extended this label fusion approach by adopting spatially varying weight-maps to capture similarity at a local level. These algorithms usually introduce a bias during label fusion when the weights are assigned independently to each atlas, allowing several atlases to produce similar label errors. These systematic (i.e., consistent across subject cohort) errors can be mitigated by taking pairwise dependencies between atlases into account during weight assignment as proposed in the Joint Label Fusion (JLF) approach (Wang et al., 2012; Yushkevich et al., 2012).

In contrast, the proposed method—Autocorrecting Walks over Localized Markov Random Field (AWoL-MRF)—pursues a different idea for tackling the label fusion problem. We hypothesize that we could achieve superior performance by mimicking the behavior of the manual rater, since virtually all segmentation methods use manual labels to define the gold-standard. Consequently, the label fusion objective developed here comprises capturing the sequential process of manual segmentation rather than optimizing atlas library weights based on similarity measure proxies and/or performing iterative inference to estimate optimal label configurations based on MRFs. Hence the novelty of the approach lies in the methodological procedure as we combine the strong prior anatomical information provided by the multi-atlas framework with the local neighborhood information specific to the given subject.

In the context of segmentation of anatomical structures such as hippocampus, the challenging areas for label assignment are mainly located at the surface regions of the structure. We observe that a manual rater traces these boundary regions by balancing intensity information and anatomical knowledge, while enforcing smoothness requirements and tackling partial volume effects. In practice, this behavior translates into a sequential labeling process that depends on information offered by the local neighborhood around a voxel of interest. For instance, a manual rater would begin by marking a boundary of a structure that they believe to be correct (high-confidence) based on anatomical knowledge. Next, the rater would identify certain regions that require further refinement (low-confidence). Then, region-by-region (patches), the rater would perform these refinements by moving from high-confidence areas to low in a sequential manner, while taking into account the information offered by neighborhood voxels from orthogonal planes. While not all groups may use this process, this tends to be a dominant order-of-operations for those using the Display tool from the MINC toolkit. This process has been used in many publications by our group (Chakravarty et al., 2008, 2009; Winterburn et al., 2013; Park et al., 2014), and serves as intuition for the development of AWoL-MRF.

The proposed label fusion method attempts to incorporate these observations into an automated procedure and is implemented as part of a segmentation pipeline previously developed by our group (Pipitone et al., 2014). The algorithmic steps of AWoL-MRF can be summarized as follows. First based on a given multi-atlas segmentation method, we initialize the label distribution for a neuroanatomical structure to be segmented. This initial label-vote distribution is leveraged to partition the given target volume in two disjoint subsets comprising regions with high and low confidence label values based on the vote distribution at the voxels. Next we construct a set of local 3-dimensional patches comprising a certain ratio of high and low confidence voxels. The spatial dependencies in these patches are modeled using independent MRFs. Finally, we traverse these patches moving from high to low confidence voxels in a sequential manner and perform the label distribution updates based on a localized (patch-based) MRF model. We implement a novel spanning-tree method to build these ordered sequences of voxels (walks).

We provide a description and extensive validation of our approach in this manuscript, which is organized as follows. First, we describe the AWoL-MRF method and the underlying assumptions in detail. Then, we provide a thorough validation of the method for the whole hippocampus segmentation by conducting multi-fold validation over three independent datasets that span the entire human lifespan. The quantitative accuracy evaluations are performed on three datasets: (1) a subset of the Alzheimer's Disease Neuroimaging Database (ADNI) dataset; (2) a cohort of First Episode Psychosis (FEP) patients; and (3) a cohort of preterm neonates scanned early in life and at term-equivalent age. Additionally we evaluate the diagnostic utility of the method by analyzing the volume differences per disease category in the ADNI1: Complete Screening dataset. We assess the accuracy and robustness of this proposed method (source code: https://github.com/CobraLab/AWoL-MRF) by comparing it with three other approaches. Our group has recently validated the performance of MAGeT-Brain (Pipitone et al., 2014) pipeline against several other automated methods. Here, we make use of MAGeT-Brain to generate candidate labels on which variety of label fusion methods can be implemented. We first compare the performance of AWoL-MRF with the default majority-vote based label fusion used in MAGeT-Brain. In addition, we compare AWoL-MRF with STAPLE (Warfield et al., 2004) and JLF (Wang et al., 2012) label fusion methods.

## Materials and methods

### Baseline multi-atlas segmentation method

MAGeT-Brain (https://github.com/CobraLab/MAGeTbrain)—a segmentation pipeline previously developed by our group, is used as a baseline method for comparison (Pipitone et al., 2014). MAGeT-Brain uses multiple manually labeled anatomical atlases and a bootstrapping method to generate a large set of candidate labels (votes) for each voxel for a given target image to be segmented. These labels are generated by first randomly selecting a subset of target images, which is referred as a template library. Then the atlas segmentations are propagated to the template library via transformations estimated by nonlinear image registration. Subsequently, these template library segmentations are propagated to each target image and these candidate labels are fused using a label fusion method. The number of candidate labels is dependent on the number of available atlases and number of templates. In a default MAGeT-Brain configuration, the candidate labels are fused by a majority vote. In previous investigations by our group (Chakravarty et al., 2013; Pipitone et al., 2014), we observed no improvements when we used cross correlation and normalized mutual information based weighted voting (Studholme et al., 1999). For the purposes of this manuscript, candidate labels generated using MAGeT-Brain will be used to serve as the input to AWoL-MRF, STAPLE, and the default majority vote label fusion methods. The use of candidate labels is non-trivial in the case of label fusion with JLF, as this method requires coupled atlas image and label pairs as input. The permutations in MAGeT-Brain pipeline generate candidate labels totaling to *number of atlases* × *number of templates*. These candidate labels no longer have unique corresponding intensity images associated with them. The use of identical atlas (or template) library images as proxies is likely to deteriorate the performance of JLF, as it models the joint probability of two atlases making a segmentation error based on intensity similarity between a pair of atlases and the target image (Wang et al., 2012). Therefore, no template library is used during JLF evaluation. Note that even though MAGeT-Brain is used as a baseline method for the performance validation in this work, AWoL-MRF is a generic label fusion algorithm that can be used with any multi-atlas segmentation pipeline that produces a set of candidate labels.

### Proposed label fusion method: AWoL-MRF

A generic label fusion method involves some sort of voting technique, such as a simple majority or some variant of weighted voting, which combines labels from a set of candidate segmentations derived from a multi-atlas library. These voting techniques normally yield accurate performance at labeling the core regions of an anatomical structure; however, the overall performance is dependent on the structural variability accounted by the atlas library. Especially in cases where only a small number of expert atlases are available, the resultant segmentation of a target image can be split into two distinct regions - areas with (near) unanimous label votes and areas with divided label votes. The proposed method incorporates this observation by partitioning the given image volume into two subsets based on the label vote distribution (number of votes per label per voxel) obtained from candidate segmentations. Subsequently, these partitions are used to generate a set of patches on which we construct MRF models to impose homogeneity constraints in the given neighborhood spanned by each patch. Finally, the voxels in these localized MRFs are updated in a sequential manner incorporating the intensity values and label information of the neighboring voxels. A detailed description of this procedure is provided below.

#### Image partitioning

Let *S* be a set comprising all voxels in a given 3-dimensional volume. Then an image *I* comprising gray-scale intensities and the corresponding label volume are defined as:

(1) $$\begin{array}{rcl} I\left(S\right) & : & \left\{x\in S\right\}\;\to \mathbb{R} \end{array}$$

(2) $$\begin{array}{rcl} L^{j}\left(S\right) & : & \left\{x\in S\right\}\to \left\{0,\;1\right\} \end{array}$$

Thus, *L*^*j*^ represents the *j*th candidate segmentation volume comprising binary label values (background:0 and structure:1) for a given image. Then with *J* candidate segmentations, we can obtain a label-vote distribution through voxel-wise normalization.

(3) $$\begin{array}{l} V\left(S\right)=\;\frac{\sum_{j}w^{j}L^{j}\left(S\right)}{J} \end{array}$$

Where, *w*^*j*^ is the weight assigned to the *j*th candidate segmentation. Now, *V*(*S*) represents the label probability distribution over all the voxels in the given image. For an individual voxel, it provides the probability of belonging to a particular structure: *V*(*x*_*i*_) = *P*(*L*(*x*_*i*_) = 1) = 1−*P*(*L*(*x*_*i*_) = 0). Now, we split set *S* into two disjoint subsets *S*_*H*_ (high-confidence region) and *S*_*L*_ (low-confidence region) such that.

(4) $$\begin{array}{rcl} S_{H} & = & \left\{x\in S\;|V\left(x_{i}\right)>L_{T}^{0}\cup^{}V\left(x_{i}\right)>L_{T}^{1}\right\}\\ S_{L} & = & \left\{x\in S\;|\;x\notin S_{H}\right\} \end{array}$$

where, $L_{T}^{0}$ and $L_{T}^{1}$ are the voting confidence thresholds for *L* = 0 and *L* = 1, respectively. Note that in the generic majority vote scenario $L_{T}^{0}$ = $L_{T}^{1}$ = 0.5 and *S*_*L*_ collapses to an empty set. In order to identify and separate low-confidence regions, these thresholds are set at higher values (>0.5) and can be adjusted based on empirical evidence (see Section Parameter Selection). As mentioned earlier, voting distributions usually form a near consensus (uni-modal) toward a particular label at certain locations, such as the core regions of structures, and therefore these voxels are assigned to the high-confidence subset. In contrast, other areas that have split (flat) label distribution are assigned to low-confidence subset.

#### Patch based graph generation

From here on, we will refer to voxels as nodes, in keeping with graph-theory convention. The partitioning operation reduces the number of nodes to be re-labeled by a significant amount. However, considering the size of the MR images, selecting a single MRF model consisting of all *S*_*L*_ nodes and their neighbors is a computationally expensive task. Additionally, the unified model usually considers global averages over an entire structure during parameter estimation for choice of prior distributions, such as *P(intensity* | *label*), which may not be ideal in cases where local signal characteristics show spatial variability. Therefore, we propose a patch-based approach, which further divides the given image in smaller subsets (3-dimensional cubes) comprising *S*_*H*_ as well as *S*_*L*_ nodes. The subsets are created with a criterion imposing a minimum number requirement of *S*_*H*_ nodes in a given patch. This criterion essentially dictates the relative composition of *S*_*H*_ and *S*_*L*_ nodes in the patch—which is referred as the “mixing ratio” parameter in this manuscript. The impact of this heuristic method of patch generation is discussed in Section Parameter Selection. The basic idea behind this approach is to utilize the information offered by the *S*_*H*_ neighbors via pairwise interactions (doubleton clique) along with the local intensity information to update the label-likelihood of *S*_*L*_ voxels. The implemented algorithm to generate these patches is described below.

**Figure 1:**
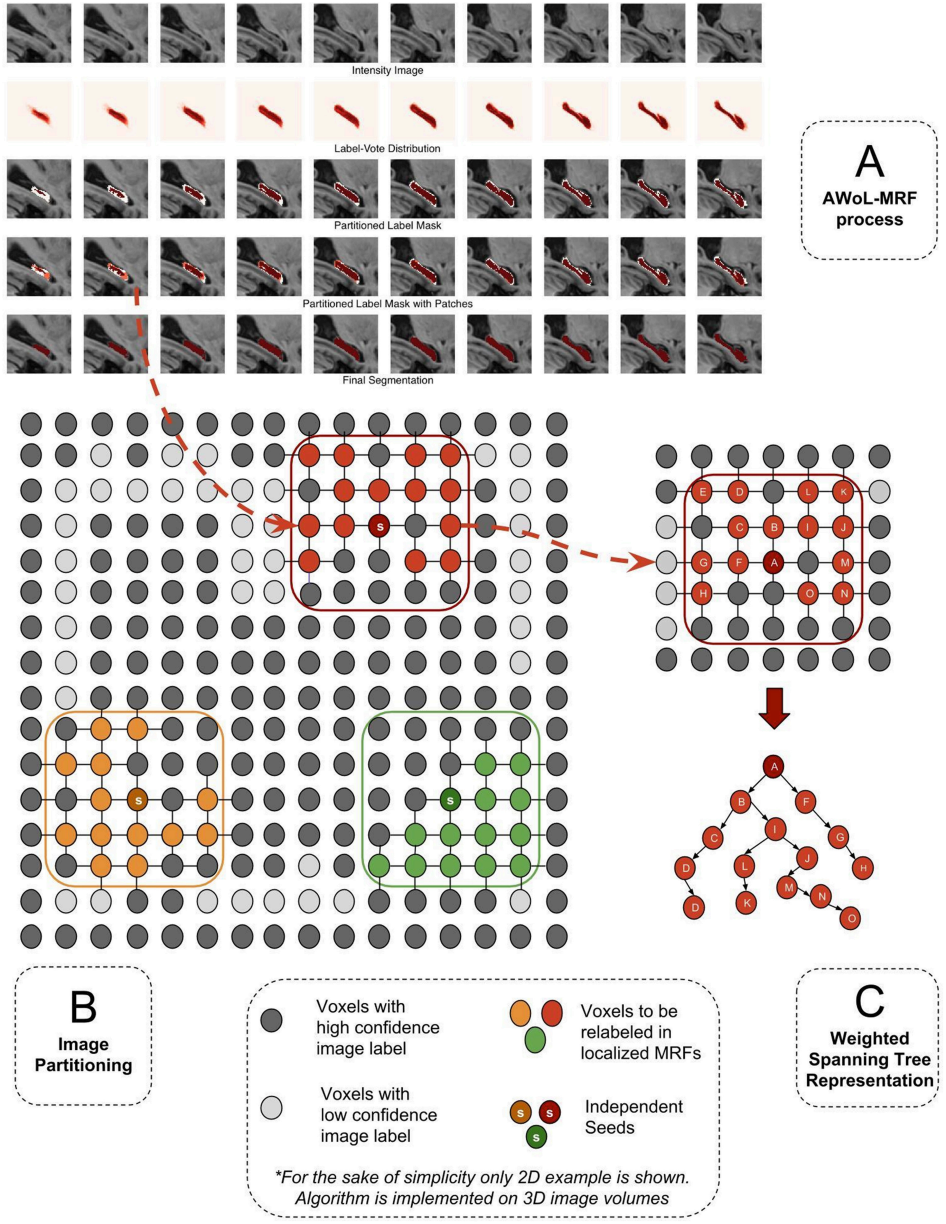
**(A)** The segmentation of a sample hippocampus in sagittal view during various stages of algorithm. Row 1: The target intensity image to be segmented. Row 2: The voxel-wise label vote distribution map for the target image based on candidate labels. Row 3: Image partitioning comprising two disjoint regions (high confidence: red, low confidence: white). Row 4: Orange Patches (localized MRFs:) comprising low confidence voxels. Row 5: Fused target labels. **(B)** Image partitioning into certain and uncertain regions and generation of patches. **(C)** Transformation of MRF graph into spanning tree representation. The tree is traversed starting from the root (seed) node and successively moving toward the leaf nodes.

First, the *S*_*L*_ nodes are sorted based on the number of *S*_*H*_ nodes in their 26-node neighborhood. Next, thresholding on the mixing ratio parameter, top *S*_*L*_ nodes from the sorted list are selected as seeds. Then, the patches are constructed centered at these seeds with pre-defined length (*L*_*patch*_). Figure 1A shows the schematic representation of the *S*_*H*_, *S*_*L*_ partitions based on initial label distribution (*V*(*S*)), as well as the overlaying patch-based subsets comprising *S*_*H*_ and *S*_*L*_ nodes. Note that depending on parameter choice (mixing-ratio and patch-length), these patches may not be strictly disjoint. In this case, the nodes in overlapping patches are assigned to a single patch based on a simple metric, such as its distance from the seed node. Additionally, these patches may not cover the entire *S*_*L*_ region. These unreachable *S*_*L*_ nodes are labeled according to the baseline majority vote. These two edge cases can be mitigated with sophisticated graph partitioning methods—nevertheless based on our exploratory investigation, such methods prove to be computationally expensive, and yield minimal accuracy improvements.

#### Localized Markov Random Field model

As seen from Figures 1A,B, the MRF model is built on nodes in a given patch (*S*_*p*_). The probability distribution associated with the particular field configuration (label values of the voxels in the patch) can be factorized based on the cliques of the underlying graph topology. With first-order connectivity assumption, we get a 3-dimensional grid topology, where each node (excluding patch edges) has six connected neighbors along the Cartesian axes. Consequently, this graph topology yields two types of cliques. The singleton clique (*C*_1_) of *S*_*P*_ is a set of all the voxels contained in that patch. Whereas the doubleton clique (*C*_2_) is a set consisting of all the pairs of neighboring voxels in the given patch. Then, for the MRF model, the total energy (*U*) of a given label configuration (*y*) is given by the sum of all clique potentials (*V*_*C*_) in this MRF model:

(5) $$\begin{array}{l} U\left(y\right)=\sum_{c\in C}V_{c}\left(y\right)=\;\sum_{i\in C_{1}}V_{C_{1}}\left(y_{i}\right)\;+\;\sum_{i,j\in C_{2}}V_{C_{2}}\left(y_{i},\;y_{j}\right) \end{array}$$

where, *y*:{*L*(*x*_*i*_)|*x*_*i*_ ∈ *S*_*p*_}. Now, assuming that voxel gray-scale intensities (*f*_*i*_ = *I*(*x*_*i*_)) follow a Gaussian distribution given the label value, we get the following relation for the singleton clique potential based on the MRF model.

(6) $$V_{C_{1}}\left(y_{i}\right)=\;\text{ log}(P\text{(}f_{i}|y_{i}))=\;-\text{log}(\sqrt{2\pi }\sigma_{y}{}_{i})-\;\frac{{\left(f-\mu_{y_{i}}\right)}^{2}}{2\sigma_{y_{i}}^{2}}$$

The mean and variance of the Gaussian model can be estimated for each patch empirically, utilizing the *S*_*H*_ nodes in the given patch as a training set. This approach proves to be advantageous especially in the context of T1-weighted images of the brain, as intensity distributions tend to fluctuate spatially. The doubleton clique potentials are modeled to favor similar labels at the neighboring nodes and are given by the following relation.

(7) $$V_{C_{2}}\left(y_{i},\;y_{j}\right)=\;-\beta \;d\left(y_{i},\;y_{j}\right)=\{\begin{array}{c} -\beta \;\;if\;y_{i}=y_{j}\\ +\;\beta \;\;if\;y_{i}\neq y_{j} \end{array}$$

The β parameter can be estimated empirically using the atlas library (Sabuncu et al., 2010). As β increases the regions become more homogeneous. This is discussed further in Section Parameter Selection. Finally, the posterior probability distribution of the label configuration can be computed using Hammersley-Clifford theorem, and is given by:

(8) $$\begin{array}{rcl} P\left(y|f\right) & = & \frac{1}{z}exp\left(-U\left(y\right)\right)\\ P\left(y|f\right)\; & \propto & \;\sum_{i\in C_{1}}\left(\log\left(\sqrt{2\pi }{\sigma_{y}}_{i}\right)+\;\frac{{\left(f-\mu_{y_{i}}\right)}^{2}}{2\sigma_{y_{i}}^{2}}\right)\\ +\;\sum_{i,j\in C_{2}}\beta d\left(y_{i},\;y_{j}\right) \end{array}$$

where *Z* is the partition function that normalizes configuration energy (*U*) into a probability distribution. The maximum *a posteriori* (MAP) label distribution is given by:

(9) $$\begin{array}{r} y^{MAP}={\text{argmax}}_{y}P\left(y|f\right)=\;{\text{argmin}}_{y}U\left(y\right) \end{array}$$

The posterior segmentation can be computed using a variety of optimization algorithms as described in the next section.

#### Inference

This section provides the details of the optimization technique used to compute posterior label distribution. Common iterative inference and learning methods such as Iterated Conditional Modes (ICM) and Expectation Maximization (EM) are computationally intensive, and ICM variants often suffer from greedy behavior that results in local optima. Here, we present an alternative approach that computes the posterior label distribution in a non-iterative, online process, minimizing computational costs. The intuition behind this approach is to mimic manual tracing protocols where the delineation process traverses from higher-confidence regions to lower-confidence regions in a sequential manner. In order to follow such a process, we transform the undirected graph structures defined by the MRF patches into directed spanning trees (see Figure 1C). Then we compute the posterior label distributions one voxel at a time as we traverse (*walk*) through the directed tree exhaustively. The directed tree structure mitigates the need for iterative inference over loops within the original undirected graph. The following is a brief outline of the implementation of the inference procedure:

1. Initialize all voxels to the labels given by the mode of baseline label distribution.
2. Transform the graph consisting of *S*_*L*_ nodes within an MRF patch into a directed tree graph, specifically a spanning tree graph with seed voxels as the root of the tree. This transformation is computed using a minimum spanning tree (MST) method (Prim's Algorithm; Prim, 1957), which finds the optimal tree structure based on a predefined edge-weight criterion. In this method, the weights are assigned based on the node adjacency and voxel intensity gradients.
  (10) $$w\left(x_{i},\;x_{j}\right)=\;\{\begin{array}{ccc} {\left(f_{i}-f_{j}\right)}^{2}\;\;if\;d\left(x_{i},\;x_{j}\right) & = & 1\\ \infty \;\;\;\;\;\;\;\;\;if\;d\left(x_{i},\;x_{j}\right) & \neq & 1 \end{array}$$
  where *d*(*x*_*i*_, *x*_*j*_) is a graph metric representing distance between two vertices.
3. Traverse through the entire ordered sequence of the MST to update the label at each voxel using Equation (9).
4. Repeat this process for all MRF patches.

## Validation experiments

### Datasets

For complete details please refer to the Supplementary Materials.

#### Experiment I: ADNI validation

Data used in this experiment were obtained from the Alzheimer's Disease Neuroimaging Initiative (ADNI) database (http://adni.loni.usc.edu/). The dataset consists of 60 baseline scans in the ADNI1: Complete 1Yr 1.5T standardized dataset (Jack et al., 2011; Wyman et al., 2013). The demographics of this cohort are summarized in Table 1. The manual segmentations for the hippocampus (ADNI-specific) were generated by expert raters following the Pruessner-protocol (Pruessner et al., 2000). These manual segmentations were used for validation and performance comparisons.

**Table 1:** **ADNI1 cross-validation subset demographics**.

	**CN (*N* = 20)**	**LMCI (*N* = 20)**	**AD (*N* = 20)**	**Combined (*N* = 60)**
Age (Years)	72.2, 75.5, 80.3	70.9, 75.6, 80.4	69.4, 74.9, 80.1	70.9, 75.2, 80.2
Sex (Female)	50% (10)	50% (10)	50% (10)	50% (30)
Education	14.0, 16.0, 18.0	13.8, 16.0, 16.5	12.0, 15.5, 18.0	13.0, 16.0, 18.0
CDR-SB	0.00, 0.00, 0.00	1.00, 2.00, 2.50	3.50, 4.00, 5.00	0.00, 1.75, 3.62
ADAS 13	6.00, 7.67, 11.00	14.92, 20.50, 25.75	24.33, 27.00, 32.09	9.50, 18.84, 26.25
MMSE	28.8, 29.5, 30.0	26.0, 27.5, 28.2	22.8, 23.0, 24.0	24.0, 27.0, 29.0

*CN, Cognitively Normal; LMCI, Late-onset Mild Cognitive Impairment; AD, Alzheimer's Disease; CDR-SB, Clinical Dementia Rating-Sum of Boxes; ADAS, Alzheimer's Disease Assessment Scale; MMSE, Mini-Mental State Examination; Values are presented as lower quartile, median, and upper quartile for continuous variables, or as a percentage (frequency) for discrete variables*.

#### Experiment II: First episode psychosis (FEP) validation

Data used in this experiment were obtained from the Prevention and Early Intervention Program for Psychoses (PEPP-Montreal), a specialized early intervention service at the Douglas Mental Health University Institute in Montreal, Canada (Malla et al., 2003). The dataset consists of structural MR images (1.5T) of 81 subjects. The demographics of this cohort are summarized in Table 2. The manual segmentations for the hippocampus were generated by expert raters following the Pruessner-protocol (Pruessner et al., 2000).

**Table 2:** **First Episode Psychosis subject demographics**.

	N^*^	***FEP (N = 81)***
Age	80	21 23 26
Gender: M	81	63% (51)
Handedness: ambi	81	6% (5)
Left		5% (4)
Right		89% (72)
Education	81	11 13 15
SES: Lower	81	31% (25)
Middle		54% (44)
Upper		15% (12)
FSIQ	79	88 102 109

Ambi, ambidextrous; SES, Socioeconomic Status score; FSIQ, Full Scale IQ. Values are presented as lower quartile, median, and upper quartile for continuous variables, or as a percentage (frequency) for discrete variables. N

* *is the number of non-missing values*.

#### Experiment III: Preterm neonatal cohort validation

This cohort consists of 22 premature neonates whose anatomical images (1.5T) were acquired at two time points, once in the first weeks after birth when clinically stable and again at the term-equivalent age (total of 44 images: 22 early-in-life and 22 term-age equivalent). The whole hippocampus was manually segmented by an expert rater using a 3-step segmentation protocol. The protocol adapts histological definitions (Duvernoy et al., 2005), as well as existing whole hippocampal segmentation protocols for MR images (Pruessner et al., 2000; Winterburn et al., 2013; Boccardi et al., 2015) to the preterm infant brain (Guo et al., 2015).

#### Experiment IV: Hippocampal volumetry

The volumetric analysis was performed using the standardized ADNI1: Complete Screening 1.5T dataset (Wyman et al., 2013) comprising 811 ADNI T1-weighted screening and baseline MR images of healthy elderly (227), MCI (394), and AD (190) patients. The segmentations were produced using 9 atlases (segmented following the Pruessner-protocol) with each method. For majority vote, STAPLE, and AWoL-MRF the number of templates was set to 19. As mentioned earlier, the use of templates is not possible with JLF due to coupling between image and label volumes from the atlas library. In the first part of analysis, we compared the mean hippocampal volume measurements per diagnosis (AD: Alzheimer's disease patients, MCI: subjects with mild cognitive impairment, CN: cognitively normal). Then in the second part of analysis, we compared the mean hippocampal volume measurements of two MCI sub-groups: MCI-converters (65 subjects converting from MCI to AD diagnosis) and MCI-stable (285 subjects with stable MCI diagnosis) within 1 year from the screening time-point. Furthermore, during both parts, we performed analysis using a linear model predictive of hippocampal volume based on diagnostic category along with “age,” “sex,” and “total-brain-volume” as covariates (data used from ADNIMERGE table from the ADNI database).

### Label fusion methods compared

We compared the performance of AWoL-MRF against MAGeT-Brain majority vote, STAPLE, and JLF. The basic approach of these label fusion methods is described below.

#### MaGeT-brain majority vote

As described in Section Proposed Label Fusion Method: AWoL-MRF, the MAGeT-Brain pipeline uses a template library sampled from the subject image pool. Consequently, the total number of candidate labels (votes) prior to label fusion equals number of atlases × number of templates. In the default MAGeT-Brain configuration, these candidate labels are fused based on simple majority vote.

#### Simultaneous truth and performance level estimation (STAPLE)

STAPLE (Simultaneous truth and performance level estimation; Warfield et al., 2004), is a probabilistic performance model that tries to estimate underlying ground-truth labels from a set of manual or automatic segmentations generated by multiple raters or methods. Note that STAPLE does not consider the intensity values from the subject image in its model comprising MRF. STAPLE carries out label fusion in an Expectation-Maximization framework and estimates performance of a manual rater or an automatic segmentation method for each label class—which is then used to find the optimal segmentation for the subject image. Software implementation of STAPLE was obtained from the Computational Radiology Laboratory (http://www.crl.med.harvard.edu/software/STAPLE/index.php).

#### Joint label fusion (JLF)

Among the modern label fusion approaches incorporating spatially varying weight-distribution, JLF also accounts for the dependencies within the atlas library (Wang et al., 2012). These dependencies are estimated based on an intensity similarity measure between a pair of atlases and a target image in a small neighborhood surrounding a voxel. This approach allows mitigation of bias typically incurred by the presence of similar atlases. Software implementation of JLF was obtained from the ANTs repository on Github (https://github.com/stnava/ANTs/blob/master/Scripts/antsJointLabelFusion.sh).

### Evaluation criteria

For experiments I, II, and III, we performed both quantitative and qualitative assessment of the results. The segmentation accuracy was measured using Dice similarity coefficient (DSC), given as follows:

(11) $$\begin{array}{l} DSC=\;\frac{2\;|A\cap^{}B|}{\left|A|+|B\right|} \end{array}$$

where *A* and *B* are the three dimensional label volumes being compared. We also evaluated the level of agreement between automatically computed volumes and manual segmentations using Bland-Altman plots (Bland and Altman, 1986). Bland-Altman plots were created with segmentations generated from 5 to 19 templates configuration. For the ADNI and FEP datasets, we performed three-fold cross validation and obtained the quantitative scores by averaging over all the validation rounds, as well as the left and right hippocampal segmentations. Constrained by the size of the Premature Birth and Neonatal dataset and the quality of certain images which caused difficulties in the registration pipeline, we simply performed a single round of validation to determine if the results that we found in Experiments I and II were generalizable to brains with radically different neuroanatomy. Due to incomplete myelination of the brain, the neonatal MR images have drastically different contrast levels. The intensity values for the hippocampus are reversed relative to T1-weighted images of the adolescent or adult human brains. These distinct attributes make it an excellent “held out sample” or “independent test-set” for performance evaluation. Thus, for this dataset, the quantitative scores are averages over left and right hippocampi over a single validation round.

Additionally, we also verified the segmentation precision using surface based metric analogous to the Hausdorff distance (Chakravarty et al., 2009). The details of this evaluation are reported in the Supplementary Materials.

## Results

### Experiment I: ADNI validation

**Figure 2:**
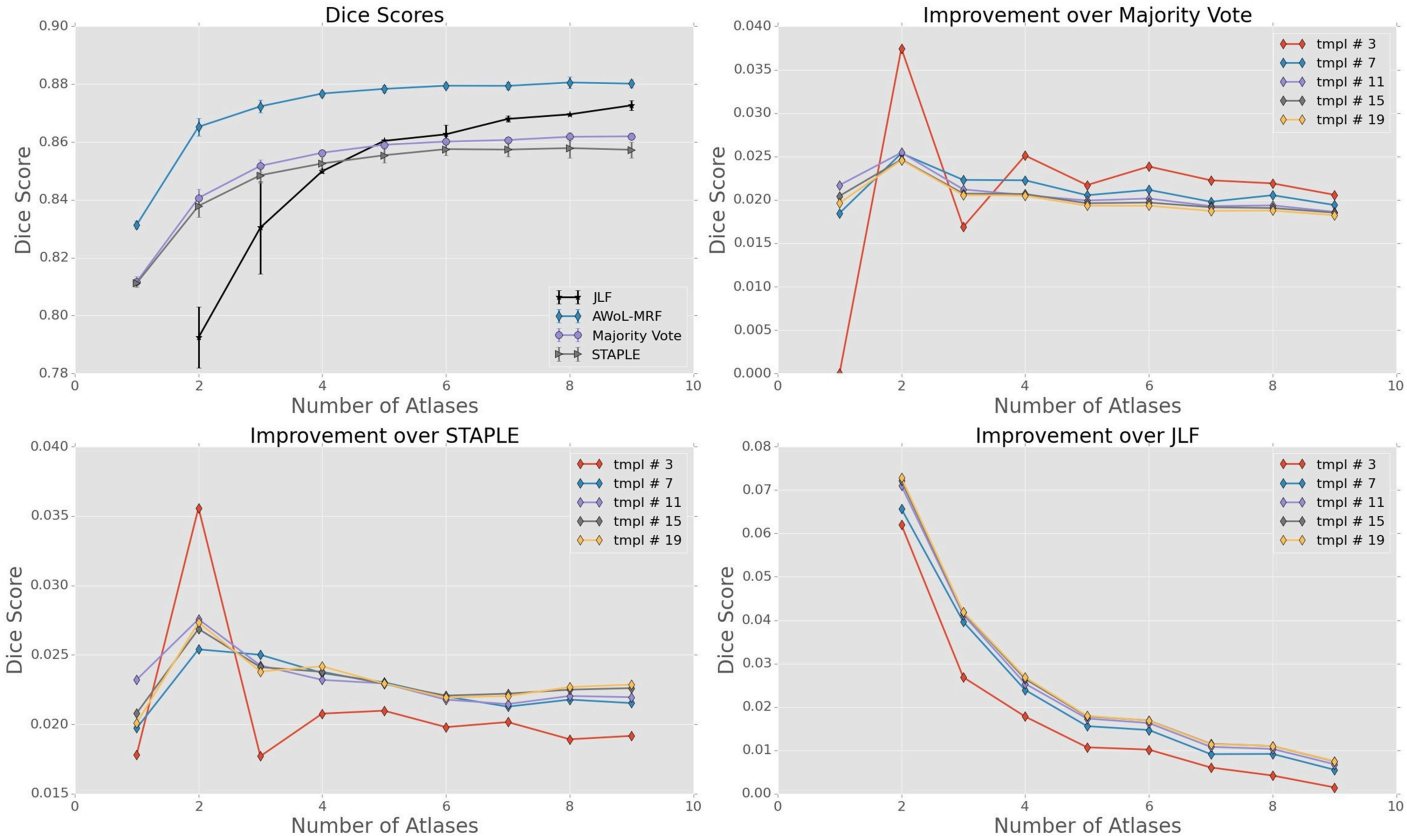
**Experiment I DSC: All results show the average performance values of left and right hippocampi over three-fold validation**. The top-left subplot shows mean DSC score performance of all the methods. Remaining subplots show the mean DSC score improvement over compared methods for different number of templates (bootstrapping parameter of MAGeT-Brain).

For ADNI dataset, the mean Dice score of AWoL-MRF maximizes at 0.881 with 9 atlases and 19 templates. As seen from Figure 2, AWoL-MRF outperforms both majority vote (0.862), STAPLE (0.858), and JLF label fusion (0.873) methods. Particularly compared to JLF, more improvement is seen with fewer atlases as AWoL-MRF reaches mean Dice score of 0.880 with only 6 atlases. The improvement diminishes with an increasing number of atlases and a smaller number of templates (bootstrapping parameter for generating candidate labels). Additionally, AWoL-MRF helps reduce the bias introduced by certain majority vote techniques while arbitrarily breaking vote-ties in the cases of even number of atlases, as previously described by our group and others (Heckemann et al., 2006; Pipitone et al., 2014). We find that AWoL-MRF corrects these decreases in performance, which is evident by the extra boosts in accuracy for the cases with an even number of atlases.

**Figure 3:**
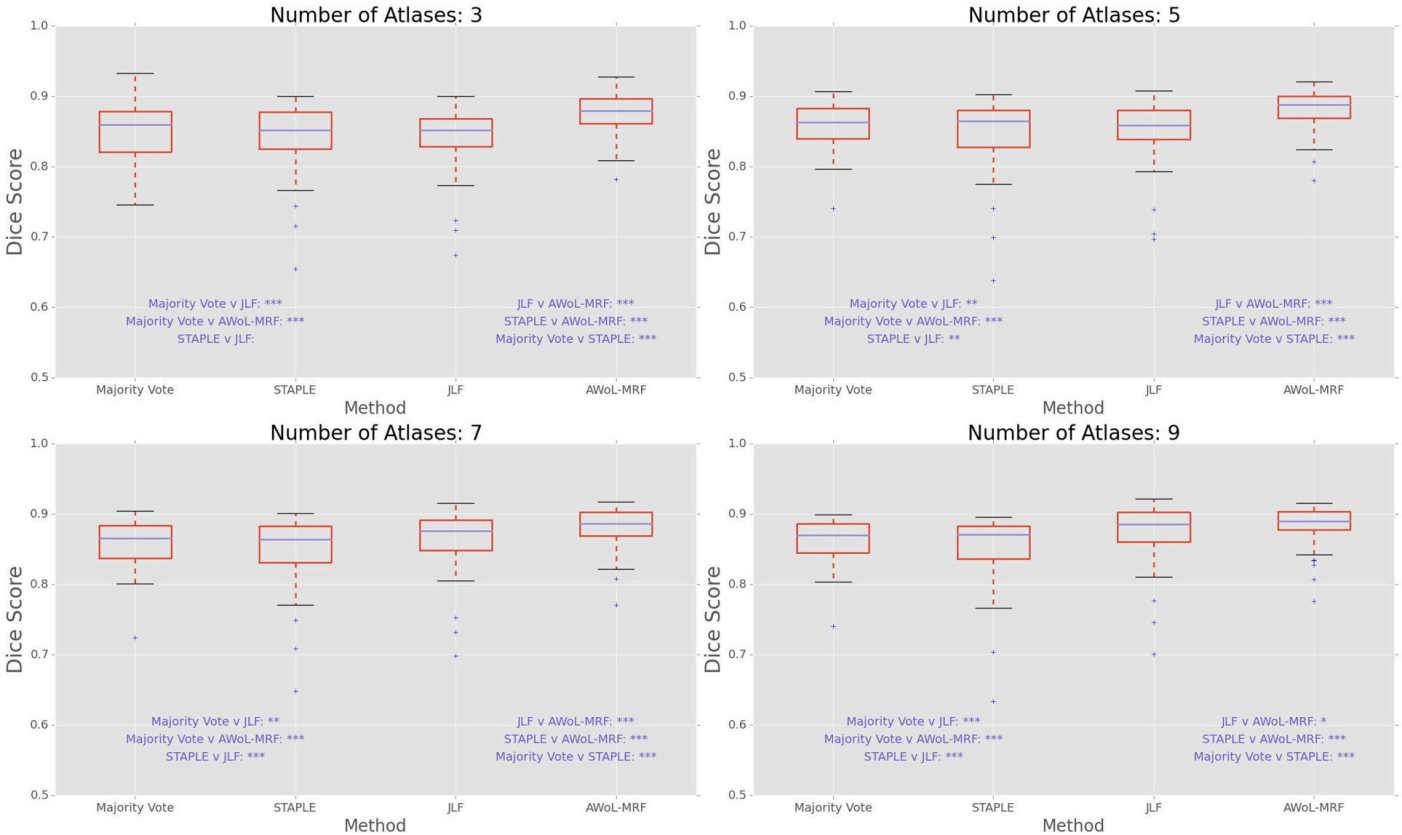
**Experiment I DSC: Statistical comparison of the performance of all methods for different atlas library sizes**. The statistical significance is reported for pairwise comparisons (^*^p < 0.05, ^**^p < 0.01, ^***^p < 0.001).

DSC distribution comparisons for a four sample configurations (number of atlases = 3, 5, 7, 9; number of templates = 11) are shown in Figure 3. These plots reveal that AWoL-MRF provides statistically significant improvement over all other methods regardless of size of the atlas library. As expected, we also notice the reduction in variance with an increasing number of atlases.

**Figure 4:**
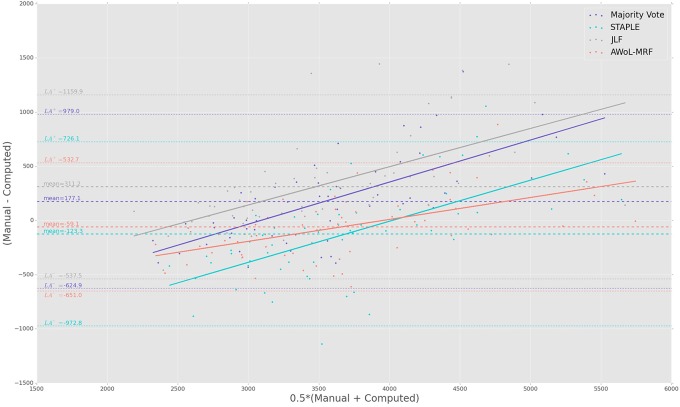
**Experiment I Bland-Altman Analysis: Comparison between computed and manual volumes (in mm^3^) for single parameter configuration of 9 atlases and 19 templates**. The overall mean difference in volume, and limits of agreement (LA+/LA−: 1.96 SD) are shown by dashed horizontal lines. Linear fit lines are shown for each method. Note that the points above the mean difference indicate underestimation of the volume with respect to the manual volume, and vice versa.

The Bland-Altman plots reveal the biases incurred with the application of each automatic segmentation method during volumetric analysis. Figure 4 shows that all four methods have a proportional bias associated with their volume estimates. Specifically, we see that in all four methods, the volumes of the smaller hippocampi are overestimated, whereas the larger hippocampi are underestimated. Nevertheless, AWoL-MRF shows the smallest magnitude of mean bias, along with tighter limits of agreement across the cohort. STAPLE displays similar mean bias values, but higher variance in volume estimation compared to AWoL-MRF, which is evident by its steeper line-slope and wider limits of agreements. Majority vote and JLF show the highest amount of positive mean bias indicating a tendency toward underestimation of hippocampal volume.

**Figure 5:**
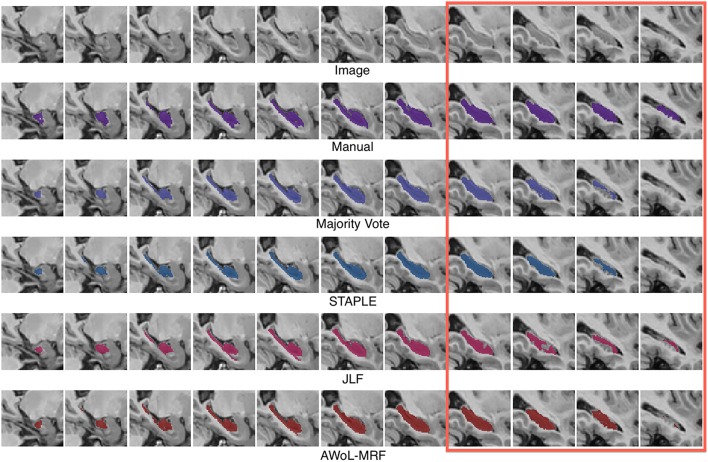
**Experiment I Qualitative Analysis: Comparison of manual vs. automatic segmentation methods**. The red rectangle illustrates a section where the superiority of the AWoL-MRF approach is particularly apparent. The segmentations are performed using 3 atlases, and the Dice scores are as follows: Majority Vote: 0.806 STAPLE: 0.833 JLF: 0.804 AWoL-MRF: 0.854. The segmentation of the left hippocampus is shown in sagittal view.

Qualitatively, improvement in segmentations is seen on the surface regions of the hippocampus. As seen in Figure 5, spatial homogeneity is improved as well.

### Experiment II: FEP validation

**Figure 6:**
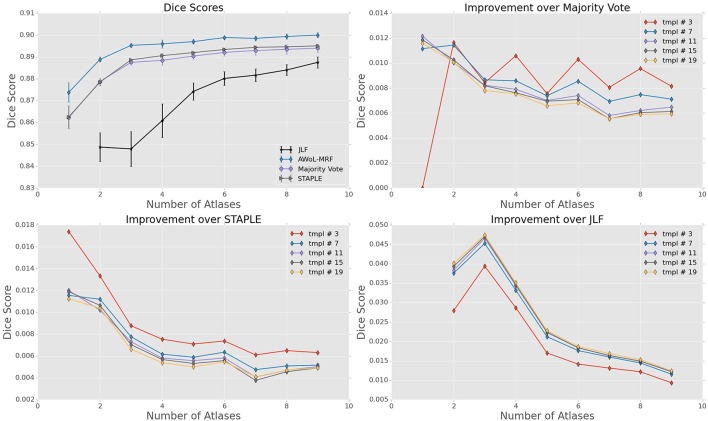
**Experiment II DSC: All results show the average performance values of left and right hippocampi over three-fold validation**. The top-left subplot shows mean DSC score performance of all the methods. Remaining subplots show the mean DSC score improvement over compared methods for different number of templates (bootstrapping parameter of MAGeT-Brain).

For the FEP dataset, the mean Dice score of AWoL-MRF maximizes at 0.897, with 9 atlases and 19 templates. Similar to Experiment I, the AWoL-MRF consistently outperforms the majority vote (0.891), STAPLE (0.892), and JLF (0.888) methods; however, the improvement is comparatively modest. More improvement is seen with fewer atlases when compared to JLF, as AWoL-MRF surpasses the mean Dice score of 0.890 with only 3 atlases (see Figure 6). The improvement diminishes with an increasing number of atlases and a smaller number of templates. In addition to a smaller atlas library requirement, the ability to reduce the bias introduced by the majority vote technique is also observed in this experiment.

**Figure 7:**
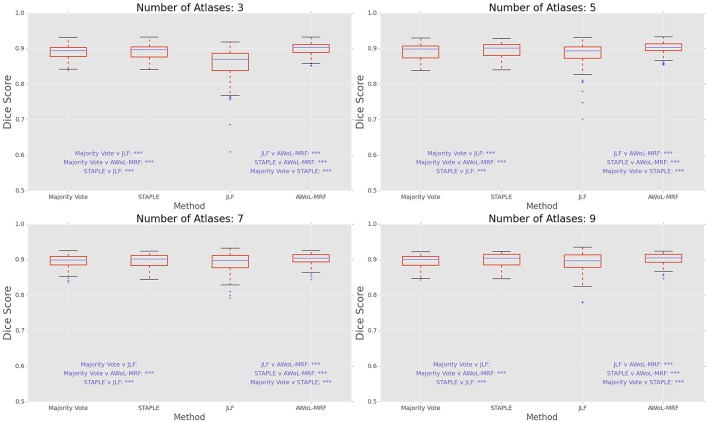
**Experiment II DSC: Statistical comparison of the performance of all methods for different atlas library sizes**. The statistical significance is reported for pairwise comparisons (^*^p < 0.05, ^**^p < 0.01, ^***^p < 0.001).

DSC distribution comparisons for four sample configurations (number of atlases = 3, 5, 7, 9; number of templates = 11) are shown in Figure 7. These plots reveal that AWoL-MRF provides statistically significant improvement over all other methods regardless of the size of the atlas library. Similar to accuracy gains, the variance of the Dice score distribution is also smaller compared to ADNI experiment.

**Figure 8:**
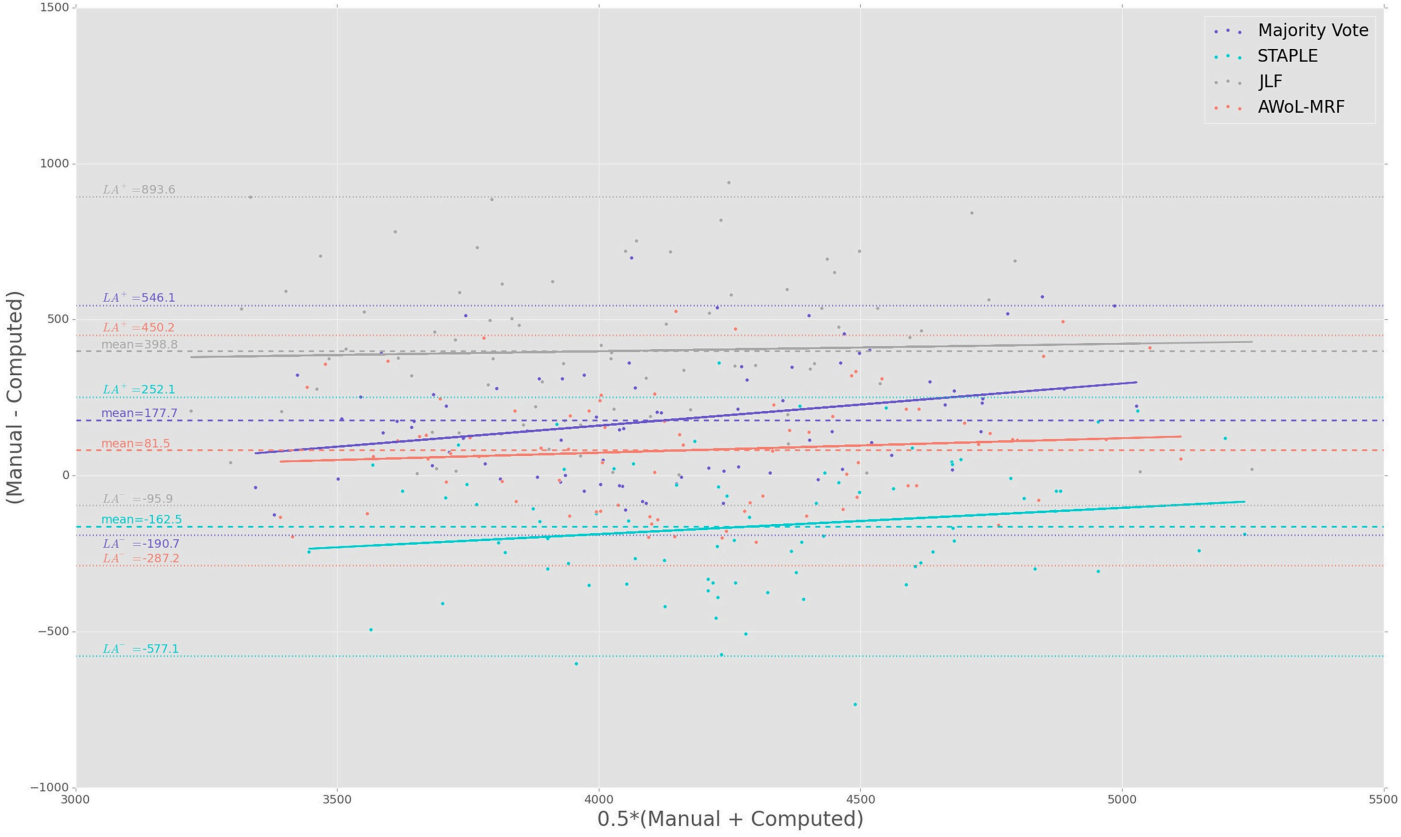
**Experiment II Bland-Altman Analysis: Comparison between computed and manual volumes (in mm^3^) for single parameter configuration of 9 atlases and 19 templates**. The overall mean difference in volume, and limits of agreement (LA+/LA−: 1.96SD) are shown by dashed horizontal lines. Linear fit lines are shown for each method. Note that the points above the mean difference indicate underestimation of the volume with respect to the manual volume, and vice versa.

The Bland-Altman plots (see Figure 8) show that both AWoL-MRF and majority vote exhibit the smallest mean proportional bias. In comparison, STAPLE and JLF show strong biases characterizing considerable overestimation (negative bias) and underestimation (positive bias) of hippocampal volume across the cohort, respectively. Quantitatively, AWoL-MRF still outperforms the other three methods, as evident from the smaller line-slope and tighter limits of agreement.

**Figure 9:**
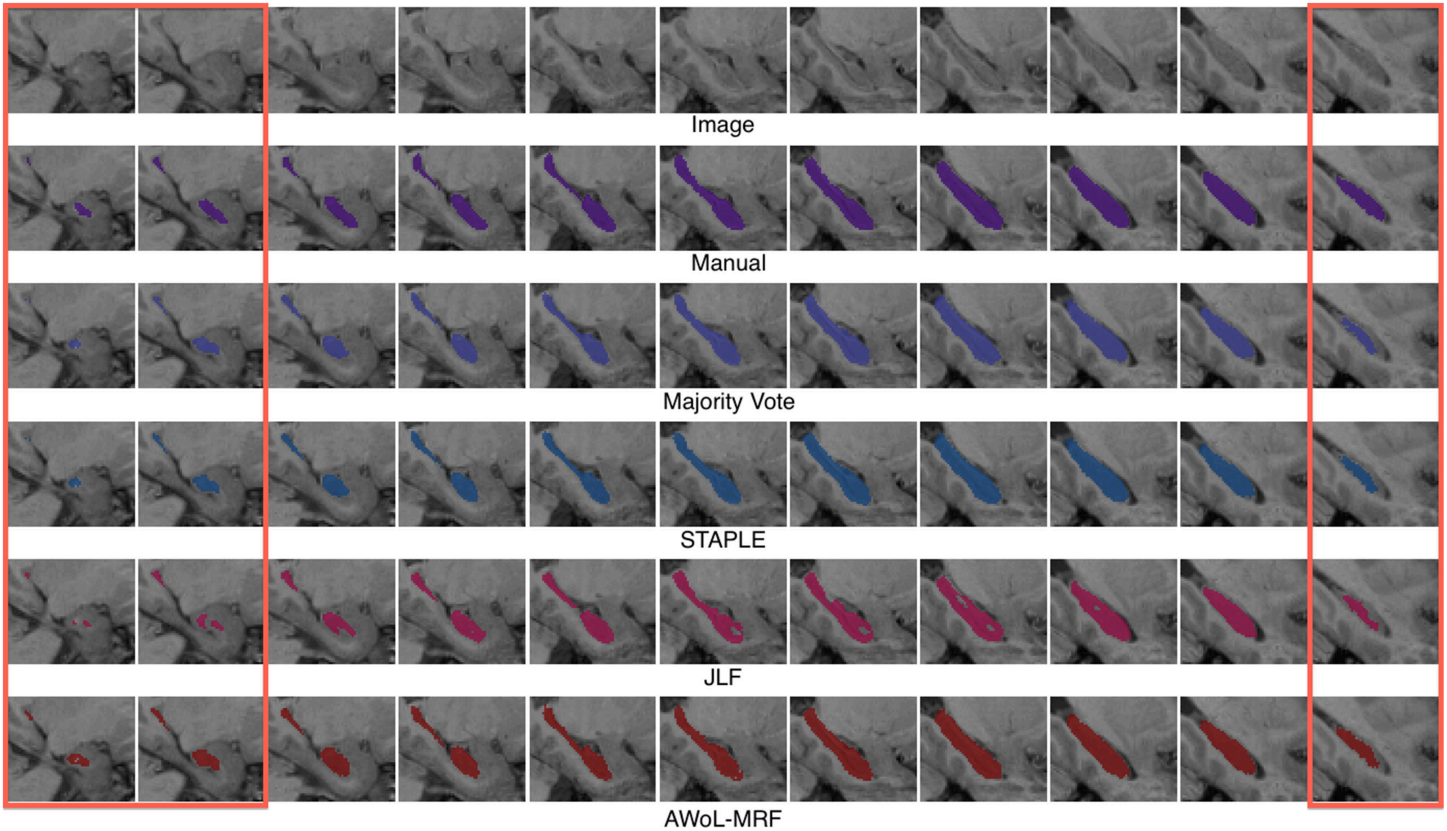
**Experiment II Qualitative Analysis: Comparison of manual vs. automatic segmentation methods**. The red rectangle illustrates a section where the superiority of the AWoL-MRF approach is particularly apparent. The segmentations are performed using 3 atlases, and the Dice scores are as follows: majority vote: 0.875, STAPLE: 0.878, JLF: 0.856 AWoL-MRF: 0.891. The segmentation of the right hippocampus is shown in sagittal view.

Similar to the ADNI experiment, qualitative improvement is seen at the surface regions of the hippocampus (see Figure 9).

### Experiment III: Preterm neonatal cohort validation

**Figure 10:**
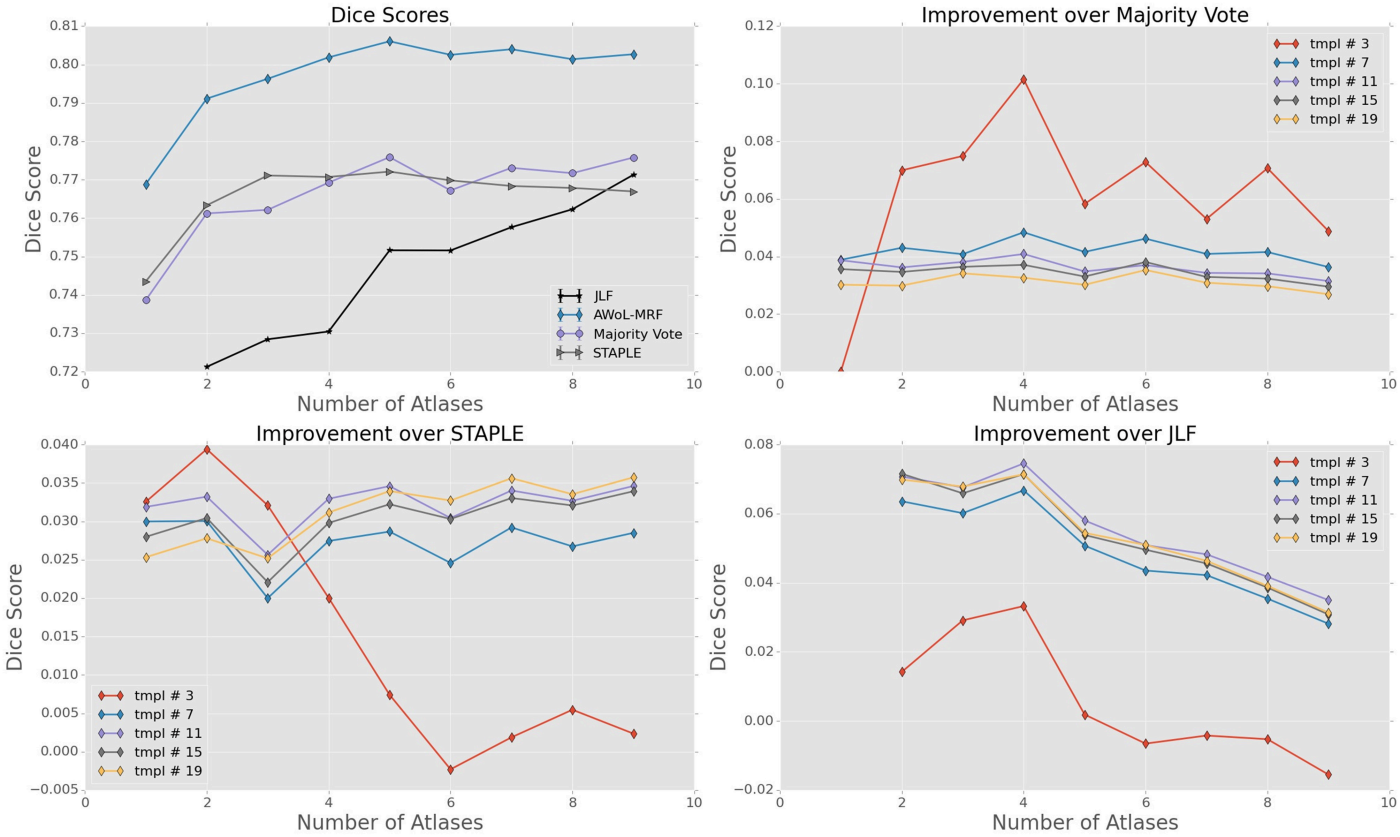
**Experiment III DSC: Preterm Neonate Cohort Validation: All results show the average performance values of left and right hippocampi over three-fold validation**. The top-left subplot shows mean DSC score performance of all the methods. Remaining subplots show the mean DSC score improvement over compared methods for different number of templates (bootstrapping parameter of MAGeT-Brain).

The mean Dice score of AWoL-MRF maximizes at 0.807, with 9 atlases and 19 templates. Similar to the first two experiments, the AWoL-MRF consistently outperforms the majority vote (0.775), STAPLE (0.775), and JLF (0.771) methods by a large amount. More improvement is seen with fewer atlases when compared to JLF, as AWoL-MRF surpasses the mean Dice score of 0.800 with only four atlases (see Figure 10). The improvement diminishes as the number of atlases increases the number of templates decreases. Also, due to the single fold experimental design for this dataset, higher performance variability is observed especially with a smaller number of templates.

**Figure 11:**
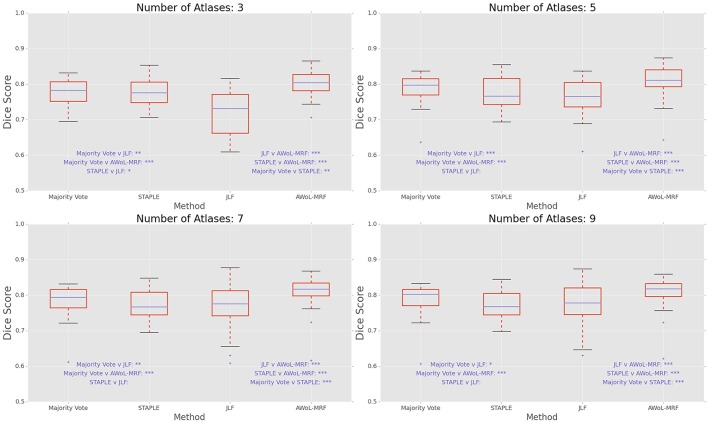
**Experiment III DSC: Statistical comparison of the performance of all methods for different atlas library sizes**. The statistical significance is reported for pairwise comparisons (^*^p < 0.05, ^**^p < 0.01, ^***^p < 0.001).

DSC distribution comparisons for four sample configurations (number of atlases = 3, 5, 7, 9; number of templates = 11) are shown in Figure 11. These plots reveal that AWoL-MRF provides statistically significant improvement over all other methods regardless of the size of the atlas library.

**Figure 12:**
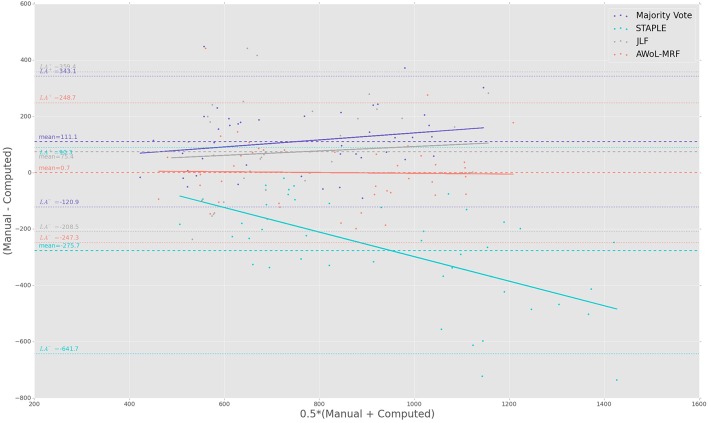
**Experiment III Bland-Altman analysis: Comparison between computed and manual volumes (in mm^3^) for single parameter configuration of 9 atlases and 19 templates**. The overall mean difference in volume, and limits of agreement (LA+/LA−: 1.96 SD) are shown by dashed horizontal lines. Linear fit lines are shown for each method. Note that the points above the mean difference indicate underestimation of the volume with respect to the manual volume, and vice versa.

The Bland-Altman plots show that both AWoL-MRF and JLF can estimate hippocampal volume with an extremely small proportional bias (see Figure 12). Compared to the ADNI and FEP datasets, the magnitude of the bias is significantly lower, with AWoL-MRF producing the best result. In comparison, majority vote consistently underestimates and STAPLE consistently overestimates hippocampal volumes across the cohort.

**Figure 13:**
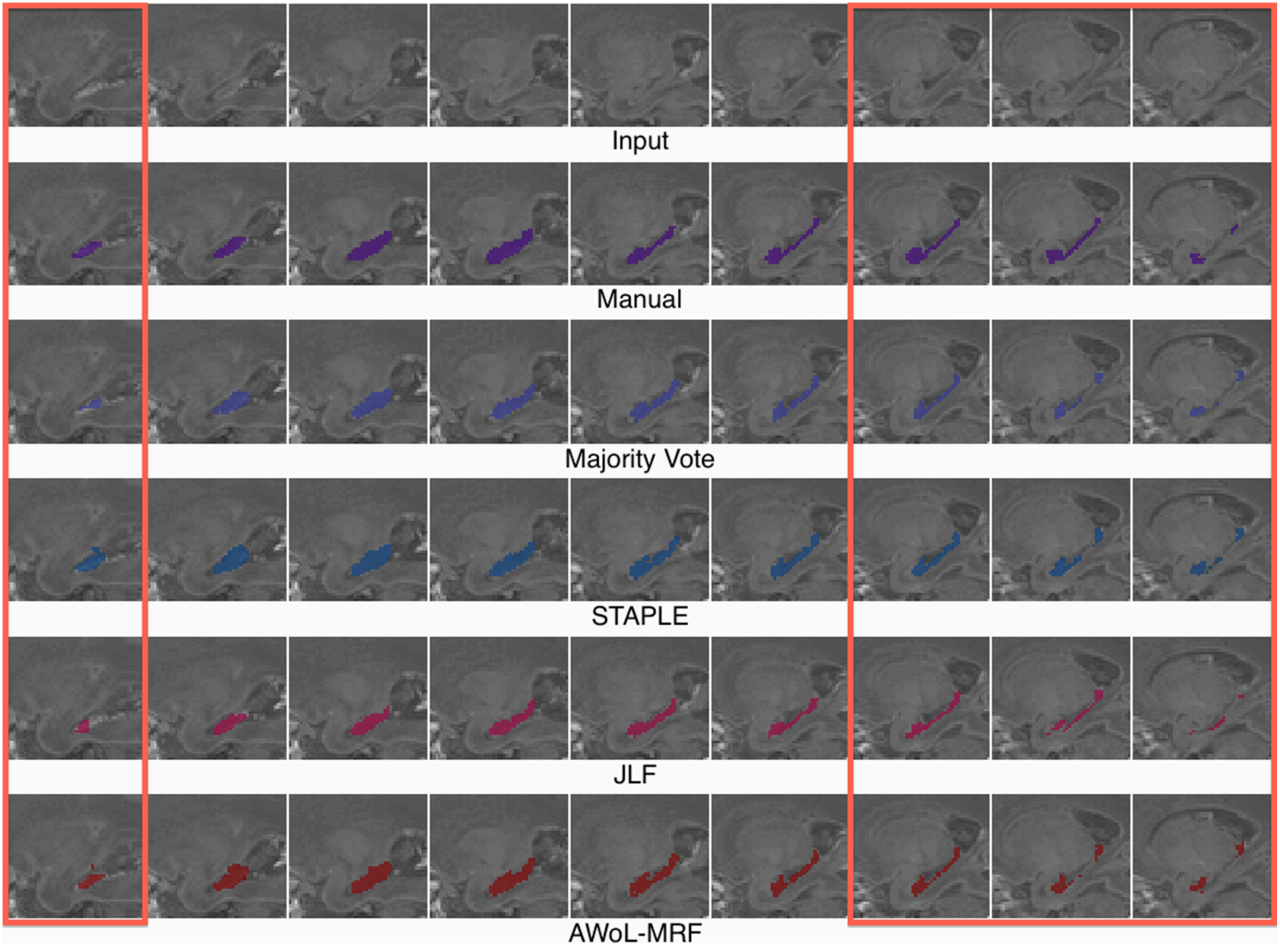
**Experiment III Qualitative Analysis: Comparison of manual vs. automatic segmentation methods**. The red rectangles illustrate sections where the superiority of the AWoL-MRF approach is particularly apparent. The segmentations are performed using three atlases, and the Dice scores are as follows: majority vote: 0.748, STAPLE: 0.760, JLF: 0.746, AWoL: 0.807. The segmentation of the left hippocampus is shown in sagittal view. Note that for this particular dataset, the brain structures are mostly unmyelinated causing a reversal of the intensity values for the hippocampal structure—as shown in the top row.

Similar to the previous two experiments, qualitative improvement is seen at the surface regions of the hippocampus (see Figure 13). Note that the intensity values for hippocampus are reversed due to incomplete myelination.

### Experiment IV: Hippocampal volumetry

#### Group comparisons between CN, MCI, and AD

**Figure 14:**
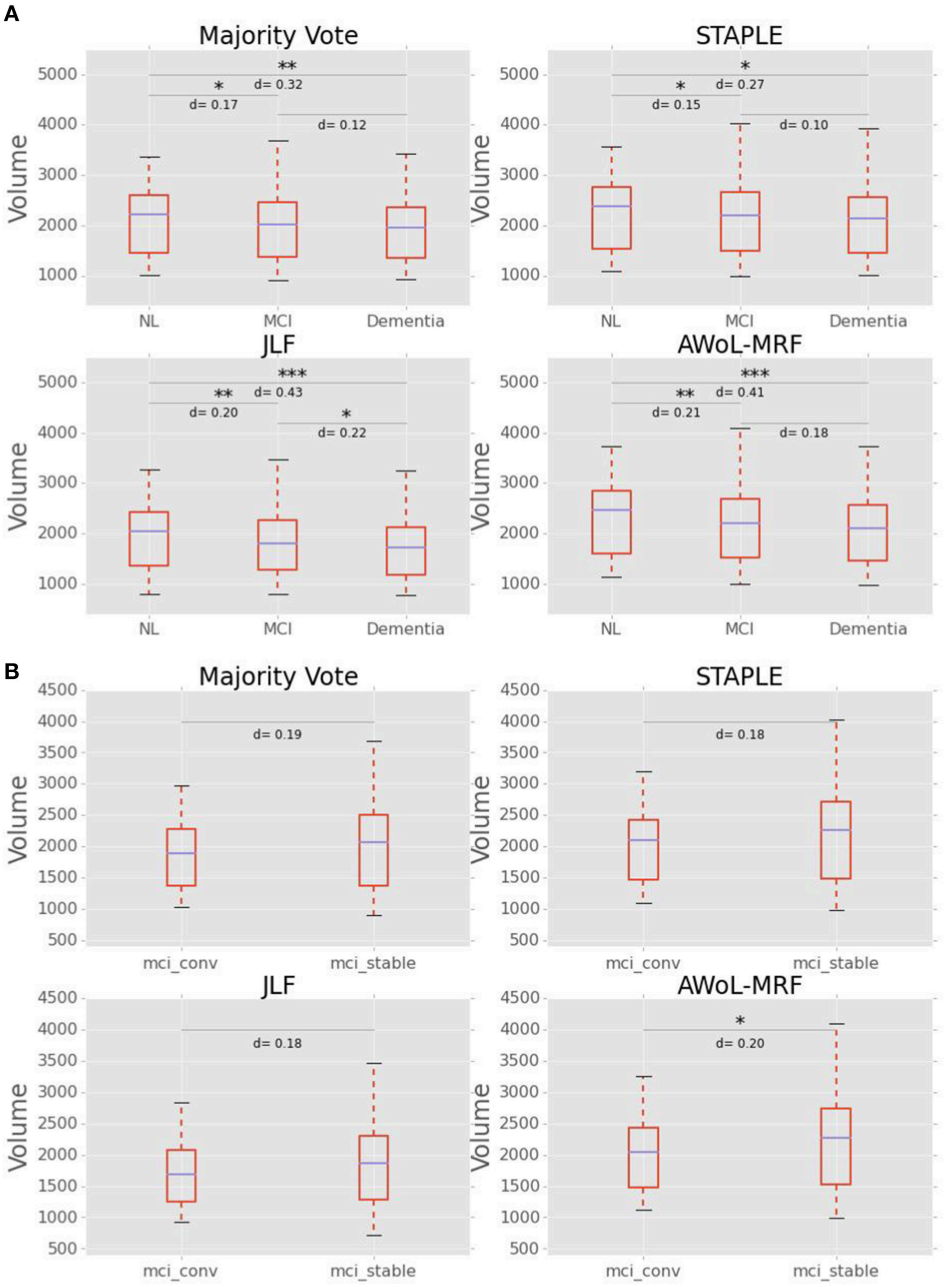
**(A)** Hippocampal volume (in mm^3^) vs. diagnoses (NL vs. MCI vs. AD). Cohen's d scores (effect size) and statistical significance is reported for pairwise comparisons between diagnostic groups. **(B)** Hippocampal Volume (in mm^3^) vs. MCI subgroups (converters vs. stable). Cohen's d scores (effect size) and statistical significance is reported for pairwise comparisons between groups.

As seen from Figure 14A, mean volume decreases with the severity of the disease for all methods. The volumetric statistics are summarized in Table 3. Based on Cohen's d metric as a measure of effect size, we see the largest separation between “CN vs. AD” diagnostic categories, followed by “CN vs. MCI” categories, and lastly between “MCI vs. AD” categories. The results show that the effect sizes are most pronounced in AWoL-MRF and JLF in all pairwise comparisons. All four methods show strong volumetric differences (*p* < 0.001 or *p* < 0.01) between “CN vs. AD” categories followed by “CN vs. MCI,” which show relatively weaker differences. JLF also shows volumetric differences between “MCI vs. AD” categories with a much weaker significance level (*p* < 0.05) compared to the other two pairwise comparisons. In the linear model analysis, all four methods show significant differences (*p* < 0.001 or *p* < 0.01) only between “CN vs. AD” and “CN vs. MCI” comparisons.

**Table 3:** **Hippocampal Volumetry Statistics of ADNI1: Complete Screening 1.5T dataset per diagnosis [subjects with Alzheimer's disease (AD), subjects with mild cognitive impairment (MCI), healthy subjects/cognitively normal (CN), as well as, MCI-converters and MCI-stable subgroups]**.

**Volumetric statistics: CN vs. MCI vs. AD Comparisons**									
	**CN**			**MCI**			**AD**		
**Method**	**Mean**	**Std. dev**	**Range**	**Mean**	**Std. dev**	**Range**	**Mean**	**Std. dev**	**Range**
Majority Vote	2084.7	615.3	[1010.0, 3364.0]	1960.5	599	[901.5, 3685.5]	1897.2	582.3	[940.0, 3422.0]
STAPLE	2236.6	659	[1097.5, 3557.0]	2124.2	649.3	[988.0, 4026.5]	2068.2	655.4	[1008.5, 3936.0]
JLF	1943.6	593.5	[796.0, 3280.5]	1803.3	572.6	[807.0, 3463.0]	1697.3	551.6	[782.0, 3242.5]
AWoL-MRF	2312.9	676.3	[1133.5, 3736.5]	2147.5	652	[991.0, 4094.5]	2047.7	631.3	[982.5, 3731.0]
**Cohen's d**						**Linear Model**			
**CN vs. MCI**			**CN vs. AD**	**MCI vs. AD**		**CN vs. MCI**	**CN vs. AD**		**MCI vs. AD**
Majority Vote	0.1727		0.3194	0.123		−3.875^***^	−3.662^***^		−0.402
STAPLE	0.1463		0.2688	0.1005		−3.424^***^	−3.001^**^		−0.101
JLF	0.202		0.4343	0.2155		−4.195^***^	−4.884^***^		−1.451
AWoL-MRF	0.2092		0.4111	0.1783		−4.424^***^	−4.657^***^		−0.987
**Volumetric Statistics: MCI-converters vs. MCI-stable Comparisons**									
**MCI-converters**						**MCI-stable**			
**Method**	**Mean**		**Std. dev**	**Range**		**Mean**	**Std. dev**		**Range**
Majority Vote	1846.2		489.6	[1036.0, 2980.0]		1995.7	619.3		[901.5, 3685.5]
STAPLE	2000.7		542.8	[1093.0, 3205.5]		2163.6	668.0		[988.0, 4026.5]
JLF	1686.8		483.9	[932.5, 2831.0]		1842.2	586.3		[807.0, 3463.0]
AWoL-MRF	2007.4		534.6	[1115.0, 3251.0]		2186.6	672.6		[991.0, 4094.5]
**MCI-converters vs. MCI-stable: Cohen's d**						**MCI-converters vs. MCI-stable: Linear Model**			
Majority Vote	0.185					−1.708			
STAPLE	0.181					−1.616			
JLF	0.192					−1.844			
AWoL-MRF	0.204					−1.965^*^			

Effect sizes of pairwise differences between groups are based on Cohen's d metric. All t-values and significance levels from a linear model comprising “Age,” “Sex,” and “total-brain-volume” as covariates. (

* *p < 0.05*,

** *p < 0.01*,

*** *p < 0.001)*.

#### Group comparisons between MCI-converters and MCI-stable cohorts

Figure 14B shows that the MCI-converters have relatively smaller volumes compared to MCI-stable group. The volumetric statistics are summarized in Table 3. AWoL-MRF shows statistically significant (*p* < 0.05) volumetric differences between these two groups, with strongest effect size based on Cohen's d metric. In the comparison using a linear model, AWoL-MRF continues to show significant volumetric differences (*p* < 0.05) between these two groups.

### Parameter selection

We studied the impact of parameter selection on the performance of AWoL-MRF with joint consideration of the segmentation accuracy and computational cost. The four parameters that need to be chosen *a priori* are: confidence thresholds ($L_{T}^{0}$ and $L_{T}^{1}$, patch-length (*L*_*patch*_), mixing-ratio (_*S*_*H*_∕*S*_*L*_)*patch*_, and the β parameter of the MRF model. Recall that the Gaussian distribution parameters in the MRF are estimated for each patch automatically using the *S*_*H*_ nodes in the given patch.

First, the confidence threshold parameters are heuristically derived from the voting distribution. As mentioned before, both *L*_*T*_^0^ and *L*_*T*_^1^ values need to be greater than 0.5 to produce non-empty low-confidence voxel set. Based on the assumptions that the high-confidence region (*S*_*H*_) comprises more structural voxels (*L*(*x*_*i*_) = 1) than the total number of voxels in the low-confidence region (*S*_*L*_), we define a following metric:

(12) $$\begin{array}{l} \rho =\;\frac{\left|S_{L}\right|}{\left|L\left(x_{i}\right)=1\right|} \end{array}$$

**Figure 15:**
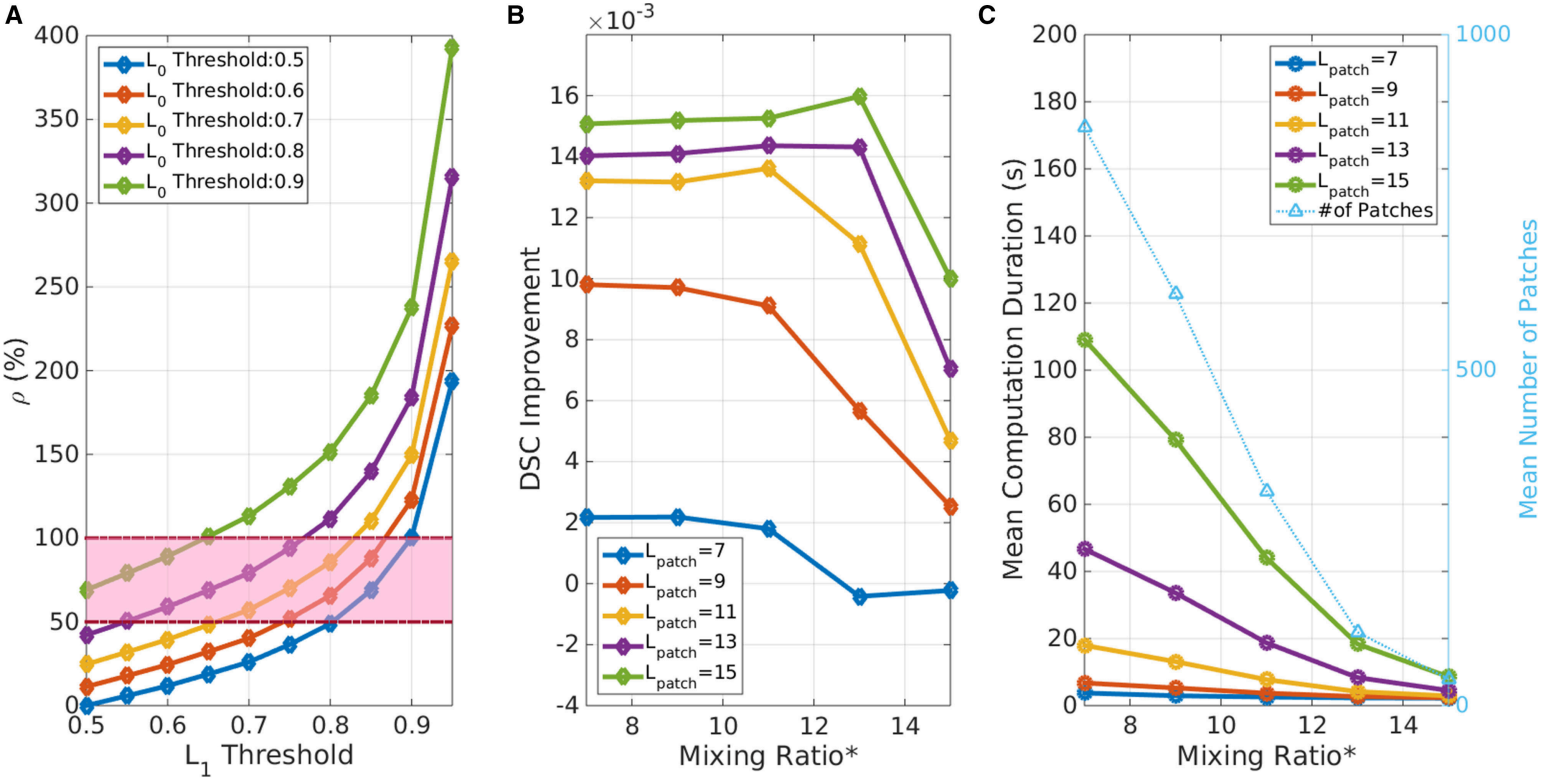
**Parameter selection. (A)** Effect of confidence threshold values on image partitioning. ρ represents the ratio of low-confidence voxels over high-confidence structural voxels. The highlighted region denotes the heuristically “good” region for threshold selection. **(B)** Effect of mixing ratio and *L*_*patch*_ on DSC performance. Mixing Ratio^*^ is the minimum required number of *S*_*H*_ nodes in the 26-node neighborhood for a given seed voxel. Note that with a larger *L*_*patch*_ performance improves. Whereas, with a smaller *L*_*patch*_ or a higher mixing ratio^*^ the performance worsens due to poor coverage over *S*_*L*_ region. **(C)** Effect of mixing ratio and *L*_*patch*_ on computational cost. The light blue line shows the number of patches for given configuration as a reference. Note that the computation time increases exponentially with a higher *L*_*patch*_ and a smaller mixing ratio^*^ (Note: mixing ratio^*^ represents the equivalent number of minimum *S*_*H*_ node requirement in the 26-node neighborhood for the seed node selections).

Then we choose confidence thresholds ($L_{T}^{0}$ and $L_{T}^{1}$), which fall in the parameter space bounded by ρ ϵ (0.5, 1). Figure 15A shows an example of these bound values—computed for the ADNI dataset in Experiment I (left hippocampus). Note that the larger threshold values imply larger *S*_*L*_ region, and consequently higher computational time. Based on this heuristic, we chose *L*_*T*_^0^ = 0.8 and *L*_*T*_^1^ = 0.6 for experiments I, II, and IV; and *L*_*T*_^0^ = *L*_*T*_^1^ = 0.7 for the experiment III.

As described in Section Validation Experiments, the patch-length and the mixing ratio parameters are interrelated and directly affect the coverage of *S*_*L*_ region. From a performance perspective, these have higher impact on the computational time than the segmentation accuracy (see Figures 15B,C). Higher *L*_*patch*_ implies larger MRF model on the sub-volume and therefore requires higher computational time. Conversely, smaller patches would reduce the computational time; but would run a risk of insufficient coverage of *S*_*L*_ region and consequently offer poor accuracy improvement. The third parameter choice of mixing ratio affects the total number of seeds/patches for a given image. A higher ratio necessitates a search for *S*_*L*_ nodes surrounded with a large number of *S*_*H*_ nodes, which reduces the total number of patches as well as the computational time. Based on the accuracy vs. computational cost trade-off analysis with respect to these parameter choices, we selected a patch-length of 11 voxels and a minimum mixing ratio of 0.0075 which translates into seed nodes surrounded by a minimum of 10 *S*_*H*_ nodes in the 26-node neighborhood, for all validation experiments.

Lastly, the β parameter of MRF model controls the homogeneity of the segmentation. It is dependent on the image intensity distribution and the structural properties of the anatomical structure. The large value of β results in more homogeneous regions giving a smoothed appearance to a structure. We selected β = −0.2 based on the results of training phase where we split the atlas pool into two groups and used one set to segment the other.

## Discussion and conclusions

In this work, we presented a novel label fusion method that can be incorporated into any multi-atlas segmentation pipeline for improved accuracy and robustness. We validated the performance of AWoL-MRF over three independent datasets spanning a wide range of demographics and anatomical variations. In Experiment I, we validated AWoL-MRF on an Alzheimer's disease cohort (*N* = 60) with median age of 75. In Experiment II, validation was performed on first episode of psychosis cohort (*N* = 81), with median age of 23. In Experiment III, we applied AWoL-MRF to a unique cohort (*N* = 22 × 2) comprising preterm neonates scanned in the first weeks after birth and again at term-equivalent age with distinctly different brain sizes and MR scan characteristics from our first two datasets. In all of these exceptionally heterogeneous subject groups, AWoL-MRF provided superior segmentation results compared to all three competing methods: majority vote, STAPLE, and JLF, based on DSC metric as well as proportional bias measurements. In Experiment IV, we validated the diagnostic utility of AWoL-MRF by analyzing the standardized ADNI1: Complete Screening 1.5T dataset. We found significant volumetric differences between “CN vs. AD” and “CN vs. MCI” groups, as well as, “MCI-converters vs. MCI-stable” groups.

In the first three experiments, we see that AWoL-MRF offers superior performance with a remarkably small atlas library, a very desirable quality in a segmentation pipeline. AWoL-MRF provides mean DSC scores over 0.880 with only six atlases (Experiment I), 0.890 with only three atlases (Experiment 2), and 0.800 with only four atlases (Experiment III) compared to other methods, which require larger atlas libraries to deliver similar performance. This is an important benefit as it reduces the resource expenditure on the manual delineation of MR images and speeds up the analysis pipelines. From a robustness perspective, we notice a reduction in the two types of biases. First, AWoL-MRF mitigates the issue of degenerating accuracy caused by the vote-ties with a small, even number of atlases. Then, more importantly, we see a consistent reduction of proportional bias, as evident by the Bland-Altman analysis.

There are several novel features that distinguish AWoL-MRF from other label fusion algorithms, particularly due to its methodological similarities to manual segmentation procedures. For instance, a manual rater estimates the voxel intensity distribution conditioned on a label class purely from the neighborhood of the target image itself and not from the atlas library. AWoL-MRF translates this into estimating the intensity distributions based on the statistics collected from the high-confidence voxels in a given localized patch in the target image. Thus, one of the key differences between AWoL-MRF and the existing multi-atlas label fusion techniques includes the decoupling from the atlas library in the post-registration stages. Once we obtain the initial label-vote distribution, we completely rely on the intensity profile of the target image and avoid any computationally expensive pairwise similarity comparisons with the atlas-library. Additionally, even though we use a commonly used MRF approach to model spatial dependencies, the novel spanning-tree based inference technique that attempts to mimic the delineation process of a manual rater differentiates AWoL-MRF from traditional iterative optimization techniques such as iterative conditional modes or Expectation-Maximization (Van Leemput et al., 2003; Warfield et al., 2004).

The key benefits of the AWoL-MRF implementation are two-fold. First we offer state-of-the-art performance using a small atlas library (< 10), whereas most existing segmentation pipelines typically make use of large atlas libraries comprising 30–80 manually segmented image volumes (Pruessner et al., 2000; Heckemann et al., 2006) that require specialized knowledge and experience to generate. Secondly, from a computational perspective, AWoL-MRF mitigates several expensive operations common among many multi-atlas label fusion methods. First, by eliminating the need for pairwise similarity metric estimation, we avoid computationally expensive registration operations that increase rapidly with the size of the atlas library. Furthermore, several extensions based on patch-based comparisons between an atlas library and a target image make use of a variant of a local search algorithm or a supervised learning approach (Coupé et al., 2011; Rousseau et al., 2011; Wang et al., 2013; Hao et al. 2014; Wu et al., 2014). For instance, Coupé et al. (2011) uses a non-local means approach to carry out label transfer based on multiple patch comparisons; Hao et al. (2014) uses a supervised machine-learning method to train a classifier using similar patches from an atlas library. Computationally these patch-based approaches, especially the implementations that incorporate non-local means, are expensive (Wang et al., 2013) and require a considerable number of labeled images (Hao et al., 2014; Wu et al., 2014). Moreover, compared to the single unified MRF models, the localized MRF model reduces the computational complexity while maintaining the spatial homogeneity constraints in the given neighborhood. It also allows the label fusion step to capture local characteristics of the image based on high-confidence regions without requiring the iterative parameter estimation and inference methods such as EM. Lastly, other confidence based label fusion methods such as Zhang et al. (2011) utilize local image appearance based metric estimated from forward and backward matching procedures involving computationally expensive k-NN search. In contrast, AWoL-MRF simply uses label-vote distribution at each voxel to compute the confidence estimate.

We believe that the performance improvements provided by AWoL-MRF can be explained by two major factors. First, we argue that the utilization of intensity values and local neighborhood constraints act as regularizers, which helps avoid over-fitting to the hippocampal model represented by the atlas library. Both majority vote and STAPLE do not consider intensity values in their label fusion stage and thus are more likely to ignore minute variations near the surface areas of the structure, which are not well represented within the atlas library. JLF, which does take intensity information into account and implements a patch-based approach, tends to perform better than majority vote and STAPLE with a relatively higher number of atlases: >4 in Experiment I and >6 in Experiment III. Therefore, we speculate that JLF is more likely to deliver superior performance in cases with larger atlas library availability, which again comes with the cost of generating manual segmentations. Second, the spanning tree based inference method tries to mimic the manual delineation process by starting with regions with strong neighborhood label information and moving progressively toward more uncertain areas. Compared to iterative methods (e.g., EM) or graph-cut based approaches (Wolz et al., 2009; Lötjönen et al., 2010) the sequential inference process may not be optimal in a theoretical sense; i.e., spanning-tree does not guarantee the global minimum for the MRF energy function. Nevertheless, we argue that the procedural similarity between the automatic and manual labeling process provides more accurate results, since the ground truth is defined by the latter.

Additionally, decoupling of label fusion process from similarity comparisons with the atlas library allows AWoL-MRF to utilize bootstrapping techniques that augment the pool of candidate labels as used by the baseline segmentation pipeline (MAGeT-Brain) in this work (Pipitone et al., 2014). Use of such techniques is not trivial with approaches using intensity information from the atlas library.

From a diagnostics perspective, the volumetric assessment of all four methods shows significant differences (*p* < 0.001 or *p* < 0.01) between “CN vs. AD” and “CN vs. MCI” groups. Consistent with the Bland-Altman analysis (see Figure 4), JLF and majority vote underestimate the volume compared to AWoL-MRF and STAPLE across all diagnostic categories. Even though the direct volumetric comparisons based on JLF yield significant differences (*p* < 0.05) between “MCI vs. AD” groups, these differences vanish in the linear model that includes “age,” “sex,” and ”total-brain-volume” as covariates. These findings are consistent with a variety of studies (Mouiha and Duchesne, 2011; Sabuncu et al., 2011; La Joie et al., 2013) highlighting the heterogeneity in hippocampal volume within MCI subjects, which results in smaller differences between MCI and AD groups. This is particularly typical in the ADNI-1 cohort MCI subjects used in this analysis, which were recently re-classified under more progressed stages of MCI or late-MCI (Aisen et al., 2010). The volumetric comparison between MCI-converters and MCI-stable groups reveals that the subjects from latter group comprise relatively larger hippocampal volumes at the screening time-point. These findings are consistent with a previous study conducted on the ADNI baseline cohort (Risacher et al., 2009). We also find that these differences remain statistically significant in the linear model that includes “age,” “sex,” and “total-brain-volume” as covariates.

A direct comparison against other methods from the current literature is difficult due to differences in the choices for gold standards, evaluation metrics, and hyper-parameter configuration, among other variables. Nevertheless, Table 4 shows a brief survey of several segmentation studies. Note that many of these studies have relied on SNT—labels provided by ADNI—for the ground-truth (manual) segmentations. A performance comparison of the baseline method based on SNT labels is discussed in our previous work (Pipitone et al., 2014), where we noticed several shortcomings of the SNT protocol (Winterburn et al., 2013; Pipitone et al., 2014), and therefore we have evaluated the presented method against the manual label based on the Pruessner protocol (Pruessner et al., 2000). Moreover, we would like to emphasize that the quality and consistency of an anatomical gold-standard is an important consideration when assessing the accuracy of an automated segmentation methodology. The Pruessner protocol used in this work reports reliabilities (Dice kappa) of 0.94 for both intra- and inter-rater over 40 subjects. Other methods (Winston et al., 2013) report the intra- and inter-rater reliability for manual segmentations to be 0.891 and between 0.82 and 0.84, respectively, using 18 subjects. Thus, depending on the choice of gold-standard for assessment of automatic methods, the expected upper bound for performance measures is likely to be different. However, the inter-rater reliabilities in Winston et al. (2013) underscore the need for a reliable segmentation methodology that is not subject to the same confounds as a manual rater in terms of consistency across raters. Our method, like many others, will always provide the same output for the automated segmentation given the same input and parameter configuration.

**Table 4:** **Summary of automated segmentation methods of the hippocampus**.

**No of atlases**	**DSCmean**	**Reference study**	**Validation**	**Dataset (ground-truth)**
9	0.881	AWoL-MRF	Three-Fold MCCV on 60 subjects	ADNI (Pruessner)
9	0.897	AWoL-MRF	Three-Fold MCCV on 81 subjects	FEP (Pruessner)
9	0.807	AWoL-MRF	One-Fold MCCV on 44 subjects	3-step segmentation protocol^b^
9	0.869	Pipitone et al., 2014	10-Fold MCCV on 60 subjects	ADNI (Pruessner)
9	0.892	Pipitone et al., 2014	Five-Fold MCCV on 81 subjects	FEP subjects
9	0.79	Guo et al., 2015	One-Fold MCCV on 44 subjects	3-step segmentation protocol^b^
30	0.82	Heckemann et al., 2006	LOOCV	Controls
21	0.862	Morra et al., 2008	LOOCV	ADNI (SNT)
55	0.86	Barnes et al., 2007	LOOCV	Controls and AD
275	0.835	Aljabar et al., 2009	LOOCV	Controls
80	0.89	Collins and Pruessner, 2010	LOOCV	Controls
30	0.885	Lötjönen et al., 2010	Segmentation of 60 subjects	ADNI (SNT)
55	0.89	Leung et al., 2010	Segmentation of 30 subjects	ADNI (SNT)
30	0.848	Wolz et al., 2010	Segmentation of 182 subjects	ADNI (SNT)
16	0.861	Coupé et al., 2011^a^	LOOCV	ADNI (Pruessner)
20	0.897 (L–HC)	Wang et al., 2012	10-Fold MCCV on 20 of 139 subjects	Landmark based semi-automatic segmentation + manual correction
	0.888 (R–HC)			
15	0.862(L–HC)	Wang et al., 2013^c^	Segmentation of 20 subjects (JLF)	BrainCOLOR
	0.861(R–HC)			
15	0.872(L–HC)	Wang et al., 2013^c^	Segmentation of 20 subjects (With corrective learning)	BrainCOLOR
	0.871(R–HC)			
9	0.841	Pipitone et al., 2014	10-Fold MCCV on 69 subjects	ADNI (SNT)

*AD, Alzheimer's Disease; MCI, Mild Cognitive Impairment; CN, Cognitively Normal; FEP, First Episode of Psychosis; LOOCV, Leave-one-out cross-validation; MCCV, Monte Carlo cross-validation; SNT, Surgical Medtronic Navigation Technologies semi-automated labels; L–HC, Left hippocampus; R–HC, Right hippocampus*.

a *AD: 0.838, MCI: n/a, CN: 0.883*.

b *See Guo et al. (2015) for manual segmentation protocol details*.

c *The method were applied in the 2012 MICCAI Multi-Atlas Labeling Challenge*.

Despite the differences in the experimental designs, comparisons with the other methods show that AWoL-MRF delivers superior performance with a significantly smaller atlas library requirement. For ADNI cohort validation, barring the ground-truth label dissimilarities, methods presented by Leung et al. (2010), Lötjönen et al. (2010) have equivalent DSC scores; however, the atlas library sizes for these methods are 30 and 55, respectively. Moreover, Lötjönen et al. (2010) use atlas selection procedure that adds another computational step to their pipeline. It may be possible that using similar number of atlases would improve our automated segmentation procedure. However, this is unlikely given the plateau effect on the number of atlases used. Moreover, to the best of our knowledge, no other study has used three drastically different datasets spanning the entire human lifespan to validate the robustness of its method. Other recent approaches (Tong et al., 2013; Zikic et al., 2014) make use of machine-learning based techniques also report similar performances. Specifically, Tong et al. (2013) make use of sparse coding and dictionary learning techniques that yield Dice scores of 0.864–0.879 depending on atlas library sizes (10–30), atlas selection, and offline training configurations. More recently, similar learning based approaches comprising sparse multimodal representations and random forests have been proposed for infant brain segmentation (Wang et al., 2014, 2015) for tissue-based classification. Nevertheless, the training phases of these methods are computationally involved requiring substantial number of atlases, making them more suitable in the context of large-scale or multimodal studies.

The computational cost of the algorithm implementation, as described in the previous section, depends on the parameter selection. From a theoretical perspective, MST transformation is the most expensive task in this method. The current implementation of MST uses Prim's algorithm with simple adjacency matrix graph representation, which requires *O*(|*V*|^2^) running time (|*V*|: number of uncertain voxels in the patch). However, this can be reduced down to *O*(|*E*|*log*|*V*|) or *O*(|*E*| + |*V*|*log*|*V*|) using a binary heap or Fibonacci heap data structures, respectively, (|*E*|: number of edges in the patch). The computational times for Experiment I with current implementation for different parameter configurations are shown in Figure 15C. The code was implemented in Matlab R2013b and run on a single CPU (Intel x86_64, 3.59 GHz). A direct computational time comparison with other methods is not practical due to hardware and software implementation differences. However, the non-iterative nature of AWoL-MRF provides considerably faster run times compared to EM based approaches, where the convergence of the algorithm is dependent on the agreement between candidate labels and can be highly variable (Van Leemput et al., 2003; Warfield et al., 2004).

In conclusion, AWoL-MRF attempts to mimic the behavior of a manual segmentation protocol in a multi-atlas segmentation framework. We validated its performance over three independent datasets comprising significantly different subject cohorts. Even though this work focuses on hippocampal segmentations, AWoL-MRF can be easily applied to other structures and scenarios with multiple label classes, which will be a part of future studies. Moreover as per the scope of this work, we only performed volumetric comparisons across groups in ADNI. While we do not perform homologous comparisons in FEP and preterm neonate cohorts, we believe the increased accuracy and precision of our method will allow us to better characterize the neuroanatomy of these groups in subsequent studies. Our validations indicate that the method delivers the state-of-the-art performance with a remarkably small library of manually labeled atlases, which motivates its use as a highly efficient label fusion method for rapid deployment of automatic segmentation pipelines.

## Author contributions

NB: AWoL-MRF algorithm development, software implementation, and experimental evaluation. JP: Software implementation of MAGeT Brain (Baseline multi-atlas segmentation pipeline). JW: Expert rater—developed Winterburn manual segmentation protocol and corresponding atlases for Experiment 3. TG, ED, and SM: Data acquisition and preprocessing for Experiment III. AV: Co-superviser of NB and JP, ML: Data acquisition and preprocessing for Experiment II. JCP: Expert rater—developed Winterburn manual segmentation protocol and corresponding atlases for Experiment I, II. MC: Superviser of NB.

### Conflict of interest statement

The authors declare that the research was conducted in the absence of any commercial or financial relationships that could be construed as a potential conflict of interest.
