# Supplementary material for: Manual-Protocol Inspired Technique for Improving Automated MR Image Segmentation during Label Fusion
Source: Front Neurosci. 2016 Jul 19;10:325. doi: 10.3389/fnins.2016.00325 (PMC4949270; doi:10.3389/fnins.2016.00325)
Supplement: Supplementary file 1 [file DataSheet1.docx]

Supplementary Material

**Manual-protocol inspired technique for improving automated MR image segmentation during label-fusion**

Nikhil Bhagwat^1,2,3*^ , Jon Pipitonec^3^, Julie L. Winterburn^1,2,3^, Ting Guo^4,5^, Emma G. Duerden^4,5^, Aristotle N. Voineskos^3, 6^, Martin Lepage^2,7^, Steven P. Miller^4,5^, Jens C. Pruessner^2, 8^, M. Mallar Chakravarty^1, 2, 7^,  and Alzheimer’s Disease Neuroimaging Initiative

*** Correspondence:** Nikhil Bhagwat, Email: nikhil.bhagwat@mail.utoronto.ca; Mallar Chakravarty, Email: mallar@cobralab.ca

# Experimental Datasets

### Experiment I: ADNI Validation

Data used in this experiment was obtained from the Alzheimer’s Disease Neuroimaging Initiative (ADNI) database (http://adni.loni.usc.edu/). The dataset consists of 60 baseline scans in the ADNI1: Complete 1Yr 1.5T standardized dataset [Wyman et al., 2013]. Twenty subjects were chosen from each diagnostic category: cognitively normal (CN), mild cognitive impairment (MCI), and Alzheimer’s disease (AD). All images were acquired using 1.5T scanners (General Electric Healthcare, Philips Medical Systems or Siemens Medical Solutions) at multiple sites using a protocol previously described in [Jack et al., 2011]. Representative 1.5T imaging parameters were TR= 2400*ms*, TI=1000*ms*, TE=3.5*ms*, flip-angle=8, field of view=240 × 240*mm*, a 192 × 192 × 166 matrix (x, y, and z directions) yielding voxel dimensions of 1.25*mm* × 1.25*mm* × 1.2*mm*. The manual segmentations for the hippocampus were generated by expert raters following the Pruessner-protocol [Pruessner et al., 2000] which is used for validation and performance comparisons. The choice of Pruessner labels was motivated from our previous validation of the baseline MAGeT Brain pipeline [Pipitone et al., 2014], in which we noted the inconsistencies in the SNT labels provided by ADNI.

### Experiment II: First Episode Psychosis (FEP) Validation

Data used in preparation of this experiment were obtained from the Prevention and Early Intervention Program for Psychoses (PEPP-Montreal), a specialized early intervention service at the Douglas Mental Health University Institute in Montreal, Canada [Malla et al., 2003]. The dataset consists of structural MRIs of 81 subjects, which were acquired at the Montreal Neurological Institute on a 1.5-T Siemens whole body MRI system. Structural T1 volumes were acquired for each participant using a three-dimensional (3D) gradient echo pulse sequence with sagittal volume excitation (repetition time=22*ms*, echo time = 9.2*ms*, flip-angle=30, 180 1*mm* contiguous sagittal slices). The rectangular field-of-view for the images was 256*mm*(*SI*) × 204*mm*(*AP*). The manual segmentations for the hippocampus were generated by expert raters following the Pruessner-protocol [Pruessner et al., 2000], which is identical to the manual segmentation protocol used in our previous validation work [Pipitone et al., 2014].

FEP data are not publicly available

### Experiment III: Preterm Neonatal Cohort Validation

This cohort consists of 22 premature neonates whose anatomical images were acquired with a specialized neonatal head coil (Advanced Imaging Research, Cleveland, OH) on a Siemens 1.5 T Avanto scanner (Erlangen, Germany) at two time points, once in the first weeks after birth when clinically stable and again at term equivalent age (total of 44 images: 22 early-in-life and 22 term-equivalency images). The 22 neonates (7 males) were born at a mean gestational age of 27.7 weeks (SD 1.9), and scanned early-in-life at 32.1 weeks (SD 1.9) and again at term equivalent age at 40.4 weeks (SD 2.1). Sequence parameters for the 3D volumetric T1-weighted images were: TR=36*ms*, TE=9.2*ms*, flip-angle=30, voxel size 1*mm* × 1.04*mm* × 1.04*mm*. The whole hippocampus was manually segmented by an expert rater using a 3-step segmentation protocol. The protocol adapts the histological definitions of [Duvernoy et al., 2005], as well as existing whole hippocampal segmentation protocols for MR images [Pruessner et al., 2000; Boccardi et al., 2013; Winterburn et al., 2013] to the preterm infant brain. This dataset was previously used by our group in the validation of an adaptation of MAGeT-Brain to the specific needs of the neonatal and prematurely born infant brain. For complete details on acquisition and manual segmentation process see [Guo et al., 2015].

Neonatal data are not publicly available.

### Experiment IV: Hippocampal Volumetry

The volumetric analysis was performed using the standardized ADNI1: Complete Screening 1.5T dataset [Wyman et al., 2013] comprising 811 ADNI T1-weighted screening and baseline MR images of healthy elderly (227), MCI (394) and AD (190) patients. (Note: The standardized ADNI1: Complete Screening 1.5T dataset consists of 818 subjects out of which seven subjects failed during the registration stage of the segmentation pipeline.)

### Surface-Distance error analysis

We performed surface distance analysis identical to the previous work [Chakravarty 2009]. The surface distance metric (M) estimates the maximum distance between the surfaces of manual and fused labels and is an approximation of symmetric Hausdorff distance. M was calculated using contour maps generated from the manual label, and 26-connected voxel erosion of the fused label. The border labels (surface) from the eroded fused-volume were intersected with manual label contour maps to compute M1 = H(a,b) and M2 = H(b,a), where H is the Hausdorff distance, then M = max(M1,M2). The results [mean (sd)] for ADNI validation (Experiment I) with 9 atlases and 19 templates are as follows:

Table 1: Experiment I surface-distance errors based on variant of Hausdorff distance. The error is measures in number of voxels, and mean and standard deviation values are reported over all the subjects in the dataset. The validation configuration comprised 9 atlases and 19 templates.

| Method | Left (# of voxels) | Right (# of voxels) |
| --- | --- | --- |
| Majority Vote | 4.6 (1.5) | 4.4 (1.1) |
| STAPLE | 4.6 (1.7) | 4.5 (1.5) |
| JLF | 5.3 (2.5) | 4.8 (2.0) |
| AWoL-MRF | 4.6 (1.9) | 4.6 (1.7) |

This preliminary analysis shows that majority vote, STAPLE, and AWoL MRF produce similar performance. In comparison JLF yields slightly higher surface-distance errors.
